# Unsupervised deep learning supports reclassification of Bronze age cypriot writing system

**DOI:** 10.1371/journal.pone.0269544

**Published:** 2022-07-14

**Authors:** Michele Corazza, Fabio Tamburini, Miguel Valério, Silvia Ferrara

**Affiliations:** 1 Department of Classical Philology and Italian Studies, University of Bologna, Bologna, Italy; 2 Departament de Prehistòria, Universitat Autònoma de Barcelona, Barcelona, Spain; Max Planck Institute for the Science of Human History, Jena, Germany, HUNGARY

## Abstract

Ancient undeciphered scripts present problems of different nature, not just tied to linguistic identification. The undeciphered Cypro-Minoan script from second millennium BCE Cyprus, for instance, currently does not have a standardized, definitive inventory of signs, and, in addition, stands divided into three separate subgroups (CM1, CM2, CM3), which have also been alleged to record different languages. However, this state of the art is not consensually accepted by the experts. In this article, we aim to apply a method that can aid to shed light on the tripartite division, to assess if it holds up against a multi-pronged, multi-disciplinary approach. This involves considerations linked to paleography (shapes of individual signs) and epigraphy (writing style tied to the support used), and crucially, deep learning-based strategies. These automatic methods, which are widely adopted in many fields such as computer vision and computational linguistics, allow us to look from an innovative perspective at the specific issues presented by ancient, poorly understood scripts in general, and Cypro-Minoan in particular. The usage of a state-of-the-art convolutional neural model that is unsupervised, and therefore does not use any prior knowledge of the script, is still underrepresented in the study of undeciphered writing systems, and helps to investigate the tripartite division from a fresh standpoint. The conclusions we reached show that: 1. the use of different media skews to a large extent the uniformity of the sign shapes; 2. the application of several neural techniques confirm this, since they highlight graphic proximity among signs inscribed on similar supports; 3. multi-stranded approaches prove to be a successful tool to investigate ancient scripts whose language is still unidentified. More crucially, these aspects, together, point in the same direction, namely the validation of a unitary, single Cypro-Minoan script, rather than the current division into three subgroups.

## Introduction

Cypro-Minoan is the term commonly used to describe a group of inscriptions dated to the latter part of the second millennium BCE, found mainly on the island of Cyprus. A dozen inscriptions also come from the port-town of Ugarit on the coast of Syria, and three have been found on Tiryns, in Greece. Currently, these inscriptions are undeciphered, which implies that the language (or languages) recorded has not been identified.

The script in which they are written is syllabic and derives directly from the Linear A script of Crete [1–4], whose status is undeciphered too. Cypro-Minoan is found on a diverse array of epigraphic supports, from tablets (though in minimal quantities), to small clay balls, metal vessels, ceramic vessel fragments and other media [5, 6]. The overall corpus counts fewer than 300 inscribed objects.

The purpose of this article is to address some outstanding problems related to the inventory of signs in the syllabary, which is still undetermined, and to reassess a long standing issue tied to the internal structure of the script itself. In light of its varied epigraphic nature, Cypro-Minoan currently stands divided into three separate, alleged sub-scripts (CM1, CM2, CM3). This implies that we do not have solid grounds to infer the homogeneous nature of the script itself. The suggestion that it may represent three different scripts has implications of linguistic division as well, as it alleges different languages being recorded. Moreover, the first proponent of the division suggested that the alleged three different scripts also recorded different languages [1], so there are linguistic implications to consider.

To shed light on this state of affairs and on the nature of the script, we have applied specific deep learning techniques, together with other traditional methods of analysis. Indeed, our approach involves a multi-stranded methodology, merging epigraphic and paleographic considerations with the application of deep neural networks. This synergy has never been applied before to any of the undeciphered scripts from the Aegean area, Linear A or the Cretan Hieroglyphic script or indeed Cypro-Minoan.

In recent years, the prominence of neural networks in different fields has advanced, thanks to the increased availability of data and progress in the methods. They have proved useful in advancing our understanding of ancient writing systems, too, to reconstruct, for instance, some documents from the Greek [7, 8] and Babylonian [9] cultures, whose content is damaged, broken or incomplete. Other approaches have applied various machine learning techniques to investigate the scribal attribution of one of the Dead Sea scrolls [10] and Linear B signs [11], identifying the hands responsible for their composition. Other approaches, however, need to be considered when dealing with scripts that are undeciphered. For example, a neural-network based approach was used to classify the presence of textual content in texts written in the Indus valley script [12], but to the best of our knowledge, no deep learning method was proposed to investigate the sign inventory of an undeciphered writing system.

To investigate Cypro-Minoan, we necessarily had to use an unsupervised deep learning technique, since the status of the sign inventory is still uncertain. The neural architecture we adopted marks the first attempt to investigate an ancient undeciphered writing system using an unsupervised method, and shows promise for further applications of neural networks in the field.

## A tripartite division

**Fig 1:**
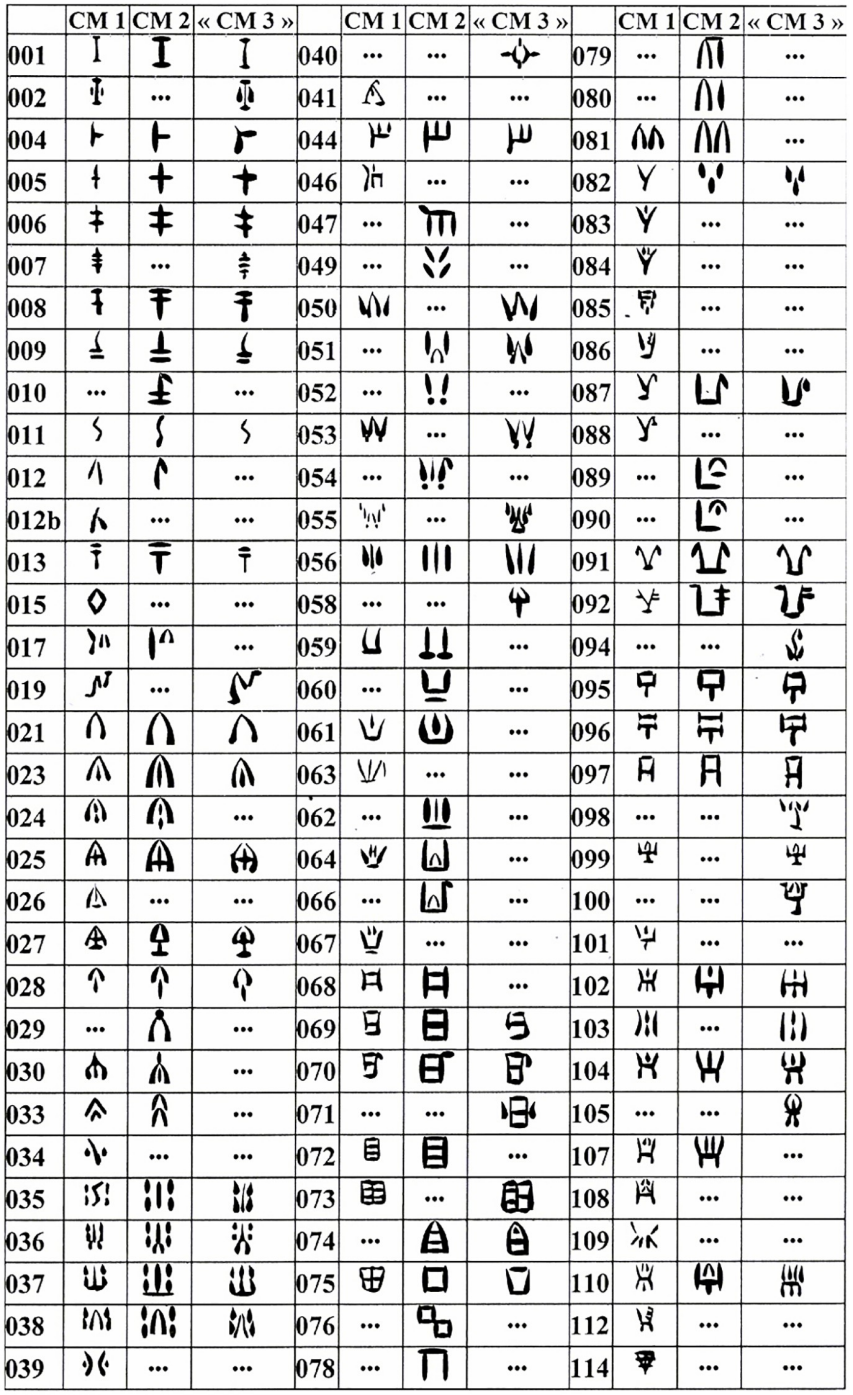
Repertoire of 96 Cypro-Minoan syllabograms and their classification in three sub-corpora according to Olivier [5].

According to the current reference corpus produced by Jean-Pierre Olivier [5], Cypro-Minoan (CM) inscriptions mainly comprise three different scripts, termed CM1, CM2, and CM3, and a total of 96 different syllabic signs in the overall inventory (Fig 1). One early inscription is thought to represent an ‘archaic’ stage of CM, closer to Minoan Linear A, termed CM0. According to this divisive classification, especially as theorized originally in 1974 [1], CM1 should include most of the inscriptions in the corpus (over 200 heterogeneous texts), ought to use the highest number of signs, and represent the main writing system on Cyprus throughout most of the Late Bronze Age (ca. 1525–1050 BCE). CM2 was considered a system derived from CM1, represented only by four clay tablet fragments from the Cypriot site of Enkomi (and no later than the 12th century BCE), therefore being of restricted use. Finally, CM3 was initially described as a second derivative of CM1, represented by some of the inscriptions from the port-town of Ugarit in Syria, and adapted to write the local Ugaritic language (although Olivier later redefined CM3 on a geographical basis as the whole set of inscriptions from Syria).

Therefore, 32 signs were supposedly shared by CM1, CM2 and CM3, while other signs were only common to two of these alleged subgroups, or even peculiar to just one (cf. Fig 1). Following the reasoning behind Masson’s original classification [1], signs unique to either CM2 or CM3 were assumed to have been adaptive innovations to write sounds of languages distinct from the language of CM1, thus explaining their absence from the latter sub-corpus. By the same token, signs absent from only CM2 or CM3 could allegedly have been discarded also for linguistic reasons.

The tripartite division of CM has been challenged in several works [2–4, 13–16]. One important criticism is that it does not fully consider the way in which different media and inscribing techniques impinge on the shapes of signs. This affects how proponents of the division have identified signs supposedly peculiar to CM2 and CM3, and afterwards interpreted such potential signs as innovations and evidence that CM2 and CM3 were scripts derived from CM1. In terms of method, to distinguish variants of the same sign (e.g. our letters Q and *q*) is a trivial task when the writing system is known. Conversely, it can be problematic when the script is undeciphered. Different signs may look very similar on different inscriptions if two hands intervene, and the opposite holds true too. With Cypro-Minoan, it has been argued that there are inconsistencies in the way signs (graphemes) have been distinguished from mere variants of the same sign (allographs).

**Fig 2:**
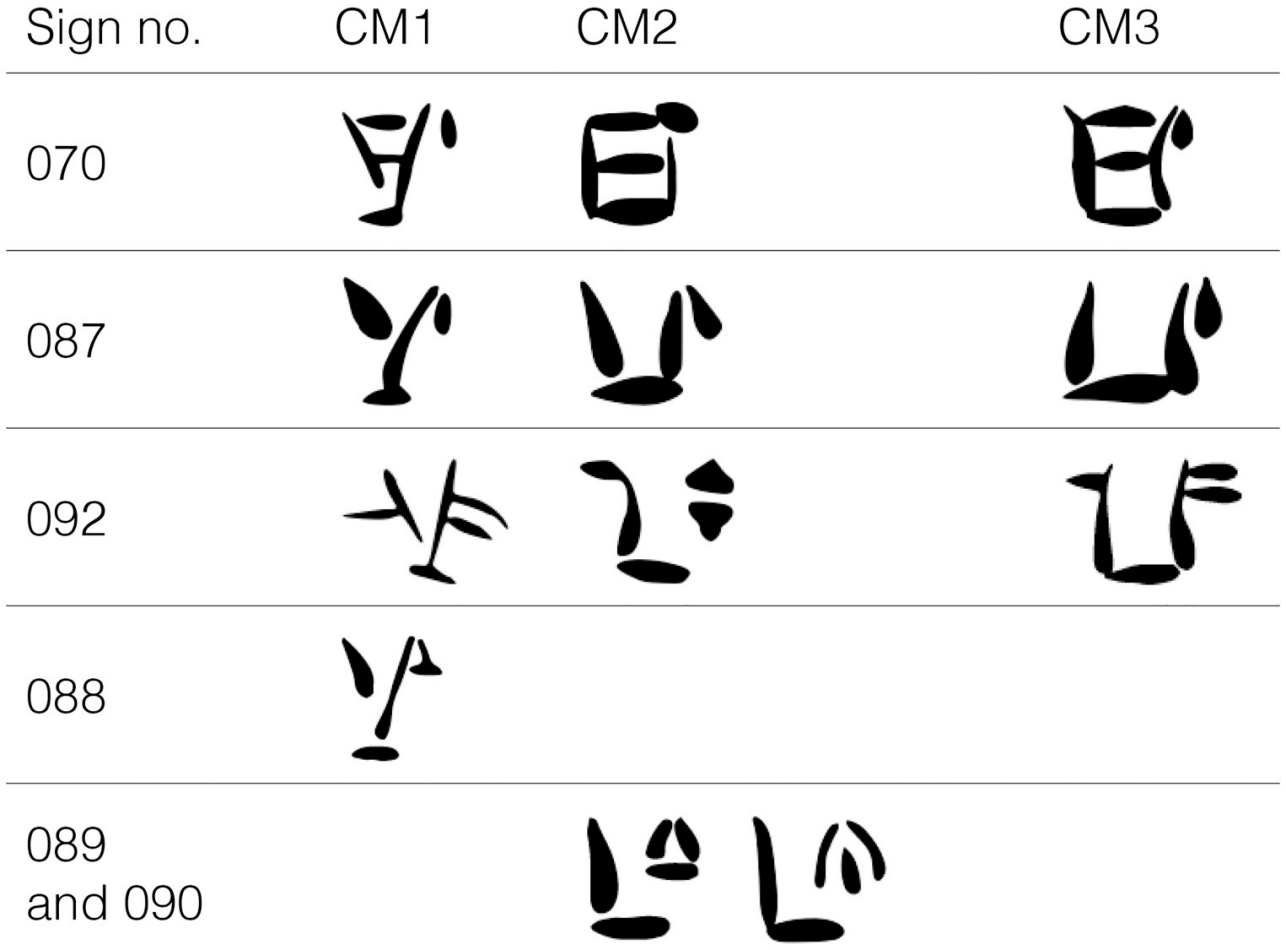
Incoherent classification of signs 070, 087 and 092 vis-a-vis 088, 089, 090 in [5] (after [3]).

This means that a sign exclusive to one CM subgroup might be just the allograph of a sign from the other subgroups. For example, in the reference editions it is recognized that certain signs (e.g. 070, 087, 092) have angular and compressed forms on clay tablets, all assigned to either CM2 and CM3, but elongated variants in various types of documents classed as CM1. Conversely, while shapes 088 (CM1), 089 (CM2), 090 (CM2) show the exact same graphic behavior, they were catalogued as three different signs (Fig 2) [3].

At the same time, other scholars have recently defended that CM2 [17] and CM3 [18] are separate scripts. Arguments in favor or against the division of Cypro-Minoan are tied to the problem of the correct identification of signs and the classification remains a debated issue. The reason is that specialists can have different views as to whether two CM sign shapes are similar or not, and therefore on whether they represent variants or distinct graphemes. Paleographic assessment remains subjective to an extent and the problem is aggravated by the lack of longer and more homogeneous inscriptions. This calls for a more neutral strategy to assess the classification of CM.

## Materials and methods

### The dataset

Our dataset contains images of signs from the CM inscriptions as published in the two reference catalogues [5, 6] and later works [3, 19–24]. This starting dataset comprised 3499 sign shapes from 230 inscriptions. This figure excludes data from 18 inscriptions without a published or usable drawing (totaling approximately 63 signs), 5 single-sign inscriptions (often considered ‘marks’ rather than writing *stricto sensu*), and 5 objects which are unepigraphic or whose status as proper inscriptions is doubted. Out of the 3499 initial signs, we removed 556 that were damaged, which meant that 10 inscriptions comprising only damaged text (= 31 sign images) were thoroughly discarded as well. As our method will consider the positional distribution of CM signs in sequences, and this data may be skewed if inscriptions written in a different script or language were included, our protocol also excluded:

- The ‘archaic’ inscription ##001 (23 signs), as it differs significantly from the rest of the corpus in terms of chronology and paleography, and 19 of its signs do not repeat [3, 25];
- 6 inscriptions (23 signs) belonging or suspected of belonging to the later Cypro-Greek syllabary (dated from ca. 1050 and onwards), which is a different writing system deciphered into a dialect of ancient Greek (##092, following [26], and ##170–172 and ##189–190, as discussed in [3, 14, 27]);

Finally, we further excluded:

- The last two signs of ##088 as their proper segmentation is debated (making the current drawings unusable) [3];

In total, after 600 sign images were excluded, we were left with a set of 2899 signs from 213 inscriptions (see S1 Table for the comparison between our dataset and the extant CM corpus). Henceforth, by “dataset” we will mean our filtered dataset, not comprising the damaged material and excluded inscriptions.

All categories of signs that have been identified in Cypro-Minoan are represented: signs used in sequences (syllabograms), two alleged logograms, numerical signs and punctuation signs. Out of the 96 categories of syllabograms established by Olivier, 95 are present in the dataset. This is because one sign shape, 083, is actually a ‘ghost sign’: its single attestation is doubtful [5] (and hence excluded here). 084 is in a similar situation, but a recent publication has wanted to see this shape in a new inscription from Erimi-*Kafkalla*, so we had to count it for our purposes. As shown by the figures reported above, our dataset comprises the majority of the extant CM corpus, and is therefore representative.

The CM signs in the dataset are represented by individual images as drawn in the published editions, which at present constitute the starting point for paleographic discussions. The digitization process used for the dataset started with a high-contrast, black and white scan of the pages of published drawings. Each sign drawing was then manually cropped, and annotated by inscription, position in the inscription, and published transcription (i.e. each sign image was assigned the sign number provided in the published editions of the inscriptions). The transcriptions follow the editions of Olivier [5] in the case of inscriptions ##002-##217 and Ferrara [6] and individual publications in the case of material edited after 2007 (which is labelled with ##ADD). Afterwards, a threshold was applied to remove noise resulting from the scan, by fitting a quadratic polynomial curve to the color histogram of the image and selecting its minimum as a threshold, so that any color with a value higher than the threshold is considered as white. Whenever artifacts from the scan could not be removed automatically, we applied the *Potrace* algorithm [28] to the cleaned images and then retraced them manually to fully match the original.

Issues with the drawings of some inscriptions in their current editions have been raised [3], but until a new corpus or dataset with revised illustrations sees the light, they continue to be the reference and starting point for all scholarly discussions on CM. Thus, we have purposefully not altered such drawings.

**Fig 3:**
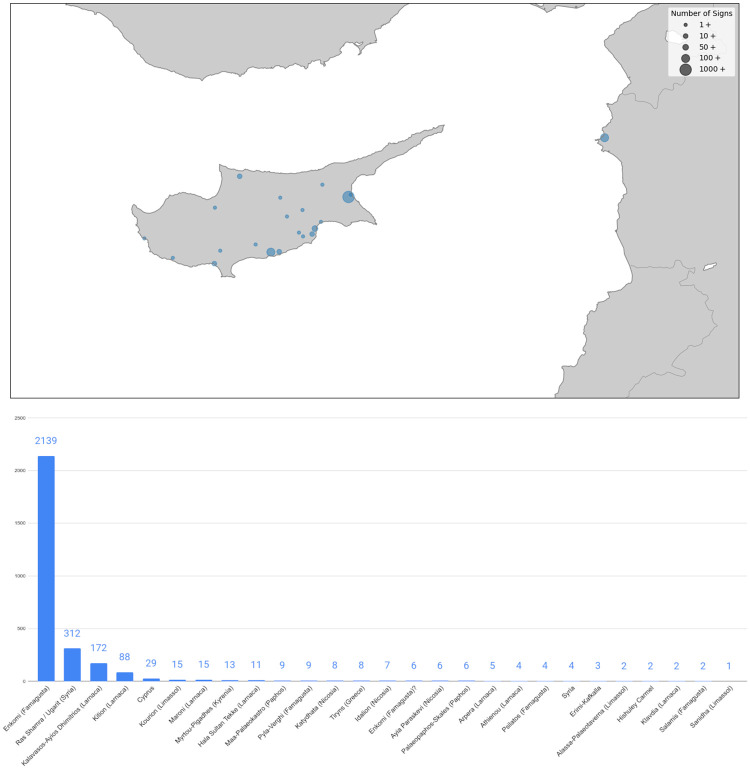
Distribution of CM signs in our dataset according to archaeological site.

It is worthwhile to underline certain properties of our CM dataset (as described above). The first is the geographic provenance of the inscriptions and the signs that make them up. Fig 3 shows the number of signs in the dataset found at each archaeological site in Cyprus and in Syria. It emerges that the largest portion of the dataset originates from the Cypriot site Enkomi, a significant amount is from *Kalavasos-Ayios* *Dhimitrios* (also on Cyprus) and from Syria, but the rest of the dataset comprises smaller amounts of material from various other Cypriot sites.

Our dataset contains 1153 signs of CM1, 1430 of CM2 and 316 of CM3. We therefore have a relatively similar amount of data for CM1 and CM2, while CM3 constitutes a smaller though not irrelevant fraction of the total number of signs. Moreover, 1732 signs are from clay tablets, while 1167 are found on other types of documents. That most CM signs are found on tablets is unsurprising, as these documents tend to contain longer texts. Still, the amount of data on other supports is an important fraction of the total.

The script as represented in the dataset shows a severe Zipf distribution, which can be observed in the frequency of the most attested signs with secure readings (as provided in the reference editions) (see Table 1). The sequence divider (|) is by far the most frequent sign, while the rest of the signs are relatively rare when compared to it.

**Table 1:** Number of attestations for the 15 most frequent signs in the dataset.

Sign	|	023	102	082	004	025	006	027	075	009	097	087	104	038	107
Attestations	466	109	89	84	74	71	65	65	63	60	57	48	44	42	42

The situation is even more problematic when we consider sign trigrams. Table 2 shows the 10 most attested sequences of three signs, of which 8 involve a divider. In fact, sign sequences that contain at least one divider constitute 57% of all sign trigrams. This has implications for our aim of building an unsupervised model that considers the positional distribution of signs in sequences, rather than just their shape. Since dividers are so prominent, any method directed towards this goal needed to use this type of sign as starting point, as we will argue in the next section.

**Table 2:** Number of attestations for the 10 most frequent sign trigrams in the dataset.

Trigram	51-28-|	|-51-28	|-102-75	|-102-35	|-38-33	4-75-|	4-87-25	4-97-|	6-82-|	9-60-59
Attestations	11	9	7	6	5	4	4	4	4	4

### DeepCluster-V2

To shed light on the inventory and classification of CM signs from an unbiased perspective, we tested the application of an unsupervised approach, whereby the model learns the relationships between signs without using any prior knowledge. Convolutional Neural Networks (CNN or Convnet) [29] represent a computational model able to carefully analyse images from different perspectives and perform different tasks on them, consistently reaching very high rates of success. As almost all neural systems dealing with images in some capacity are based on CNN [30], they seemed most fitting to our purposes. We based our model on an unsupervised method called DeepClusterv2. This model uses a CNN with residual connections (ResNet) [31], a specific kind of CNNs, to perform automatic clustering of images.

**Fig 4:**
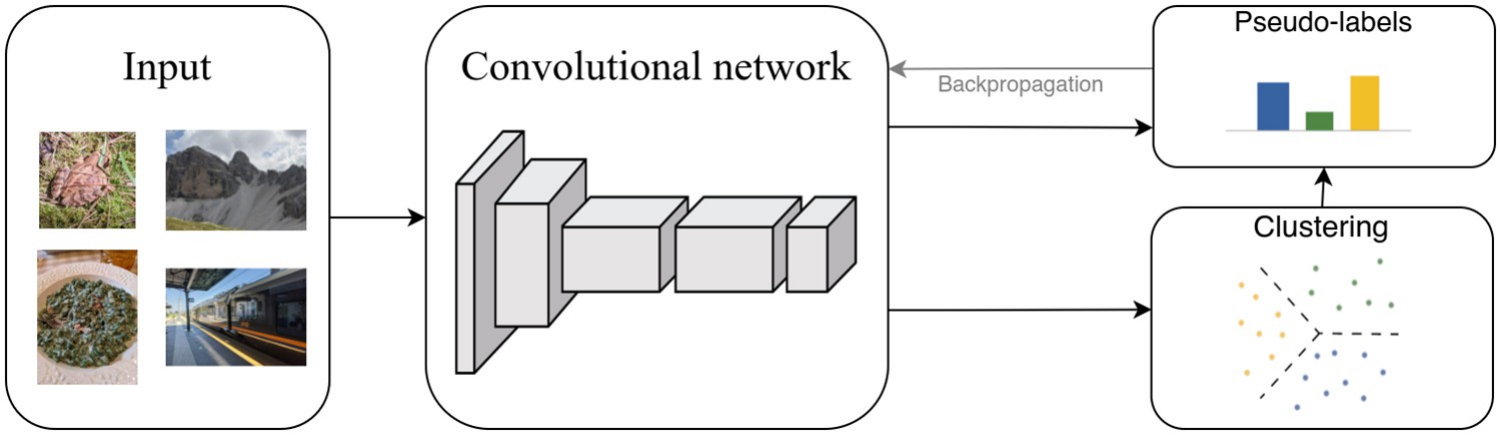
DeepCluster structure [32].

DeepCluster-v2 is based on DeepCluster [32], which uses a K-Means clustering algorithm to extract pseudo-labels from the images, which in turn are used to train a classifier over them (Fig 4). The clustering step happens on normalized (unit-norm) vectors and uses a dot product as the distance metric, which is equivalent to cosine distance on an hypersphere. The main issue of this model, however, is that the classification layer used to train the model on the pseudo-labels needs to be reinitialized at every epoch, as the assignments of images to clusters changes during training. This leads to model instability and impacts its performance. DeepCluster-v2 addresses this problem by replacing the last layer of the model with the centroids obtained from K-Means. The application of the last layer to the vectors corresponds to the dot product between the vector and each centroid. Since both the centroids and the vectors are normalized to have unit-norm, this corresponds to calculating the cosine proximity between centroids and vectors. Furthermore, data augmentation is applied to the image, in the form of random crops, color distortion and random horizontal flips of the images. This way, multiple augmented versions of the image are used to train the model. Finally, additional improvements such as a Multi Layer Perceptron (MLP) projection head and cosine learning rate schedule are added to the model.

Since no gold standard categorization exists for the signs of CM, we used a dimensionality reduction technique, namely the t-distributed stochastic neighbor embedding (t-SNE) [33], to project the high-dimensional latent space in 3D for executing a preliminary visual inspection of the results.

The use we made of this neural approach and other validation steps for the model as well as parameter selection will be discussed in the next section.

#### Unsupervised model for undeciphered scripts: *Sign2Vec_d_*

**Fig 5:**
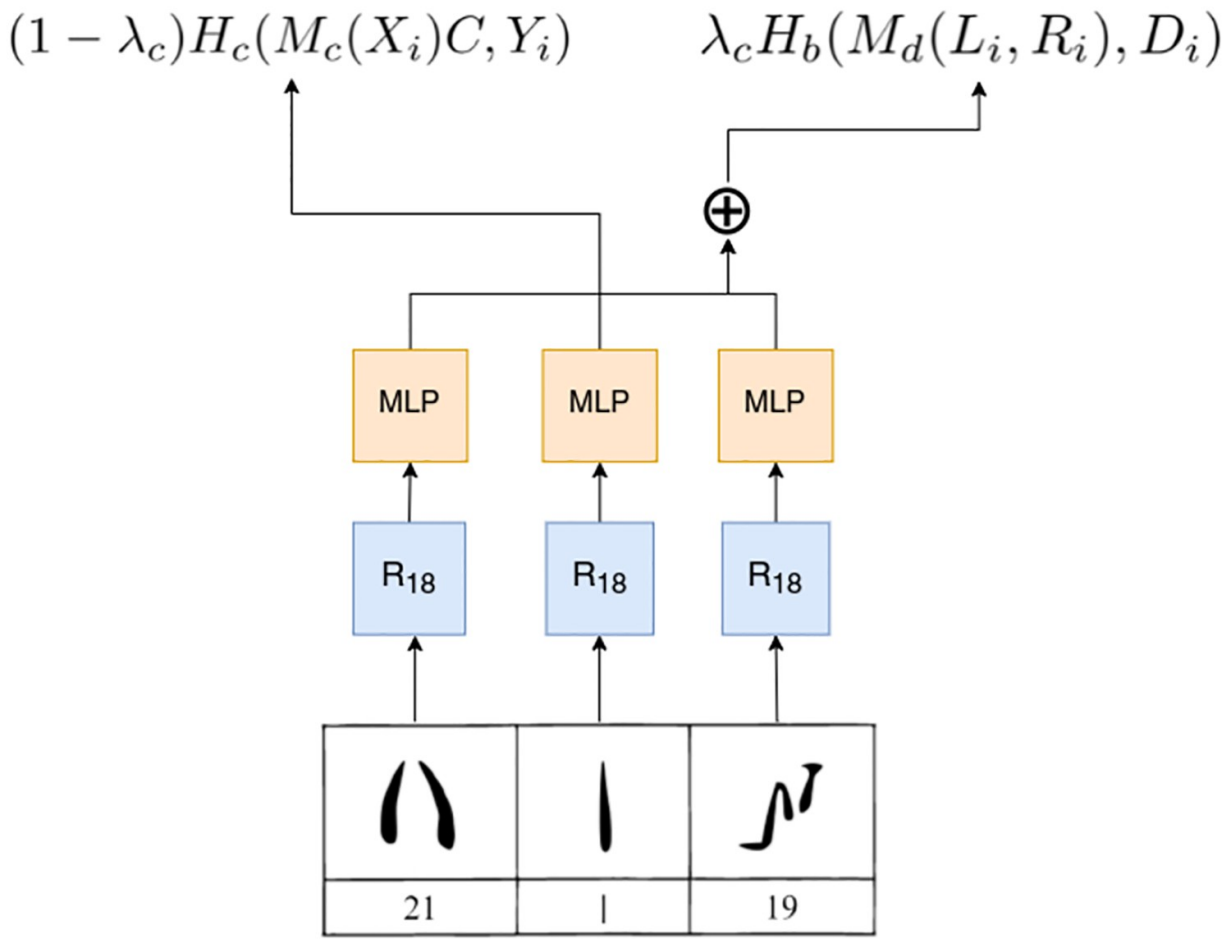
*Sign2Vec_d_* (signs drawn after [5]).

As graphic similarity between any two sign shapes is not a sufficient criterion to prove that they represent the same grapheme, we needed a method that overcame this limitation. Thus, we modified DeepCluster-v2 to be context-aware and adapted the model to our purposes. By ‘context’ we mean the collocation of any sign with respect to other signs in any string of Cypro-Minoan text. This follows the premise that any sign in a writing system is bound to occur more frequently in certain positions within a sequence (a word or phrase) by virtue of its sound value and the distribution of that sound (or sounds) in the underlying language. Thus, by deeming any two signs more closely related considering not just how similar their own shapes are, but also how much their neighboring signs resemble one another, the factor of chance resemblance is reduced. As in our dataset the number of damaged signs and the short length of many of the texts limit the amount of available contexts significantly, we leveraged an important aspect of CM: the frequent use of dividers that separate sequences. Since some signs are found predominantly in sequence-initial or sequence-final position, we taught the model to predict, based on the images on the left and right positions of a trigram, whether the central image is a divider. We named the resulting model *Sign2Vec_d_* (Fig 5). The implementation of *Sign2Vec_d_* and DeepClusterv2 used in this paper are hosted at https://github.com/ashmikuz/sign2vec_d.

For each non-damaged sign *X*_*i*_ we considered the non-damaged signs before and after it (*L*_*i*_ and *R*_*i*_) to predict whether *X*_*i*_ is a sequence divider.

Three identical ResNet CNNs analyse the signs images for producing intermediate representations and then three identical MLP networks project the outputs of the ResNets. The output obtained from the central sign (*M*_*C*_(*X*_*i*_)) is then multiplied with the centroids matrix obtained from K-Means (*C*). The concatenation of the vectors obtained from the context (*M*_*D*_(*L*_*i*_, *R*_*i*_)) is then fed to a linear layer that performs a binary classification task, to decide whether *X*_*i*_ is a divider or not.

The *Sign2Vec_d_* loss is then defined as the sum of two components: the first is the categorical cross entropy *H*_*c*_ between the cosine similarities of the latent representation of the sign and the centroids *M*_*c*_(*X*_*i*_) and the actual centroid *Y*_*i*_ obtained from K-Means. The second component is the binary cross entropy *H*_*b*_ between the linear projection of the concatenated vectors representing the left and right context images, denoted as *M*_*d*_(*L*_*i*_, *R*_*i*_) and a binary value that indicates whether the central sign between *L*_*i*_ and *R*_*i*_ is a divider. The λ_*c*_ constant is used to weight the relative importance of the two components of the loss function. In our experiments it was set to 0.7.

CM sequences found in isolation or at the beginning and end of inscriptions tend to lack dividers in at least one of their extremes. In such cases, a solution was needed to mark the context of initial and final signs. Thus, an image of a divider, selected randomly from the many separators attested in the dataset, was also imposed as the left context of an unmarked beginning or the right context of an unmarked end of sequence. When the real beginning or end of a sequence is unknown because the inscription is damaged or broken, context was instead marked by a randomly generated artificial image comprised of dots that denote damage, following the convention for the representation of damaged text in the drawings of the editions of Olivier [5]. Finally, since the contextual component of *Sign2Vec_d_* is tasked with predicting whether the central sign of a trigram is a divider, we also needed to consider the artificial dividers we inserted at the end of inscriptions. A fully black image was used to denote the end of a document whenever there was no sign after an artificial divider.

We trained 20 different models initialized with different random parameters. By applying this procedure, the model becomes less susceptible to the the random initialization of its parameters. The full model parameters are presented in S2 Table. Afterwards, we used the 20 trained models to perform statistical tests or concatenate the vector representations of the signs. We then applied the model to the CM dataset and examined the result by creating a 3D scatter plot from a t-SNE projection of the concatenated learned representation of each sign from all 20 models. This visualization suggested that our model learns a meaningful approximation of the distinction between graphemes in CM. This procedure was repeated for both DeepClusterv2 and *Sign2Vec_d_*.

## Results and discussion

The 2560-dimensional representation obtained by applying the proposed neural model, namely *Sign2Vec_d_*, and the baseline, *DeepClusterv2*, was the starting point for any further processing and paleographic consideration.

### Arrangement of signs in two paleographic subgroups

**Fig 6:**
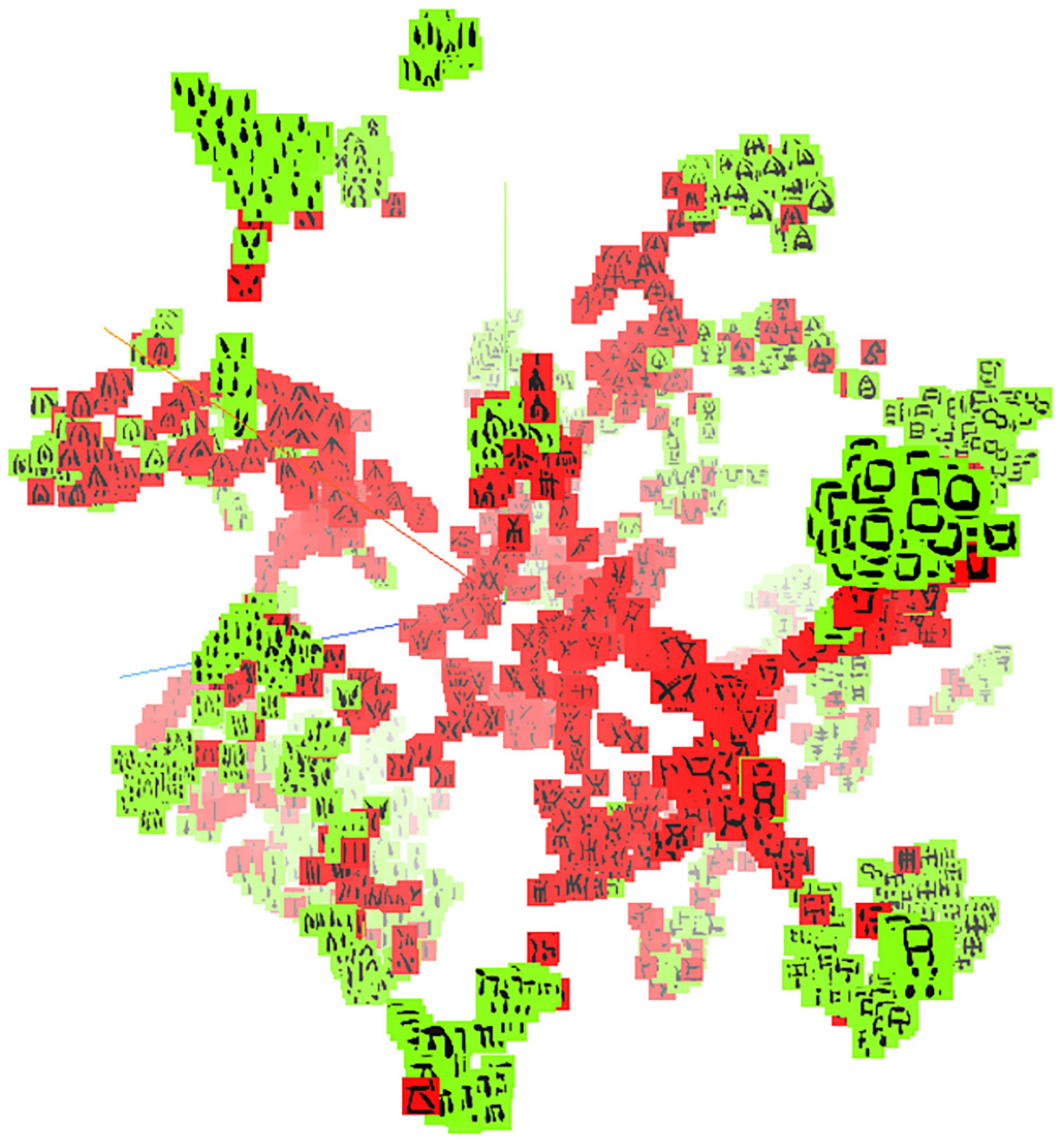
Separation of CM signs from clay tablets (in green) and signs found in other types of inscription (in red) in the 3D scatter plot.

**Fig 7:**
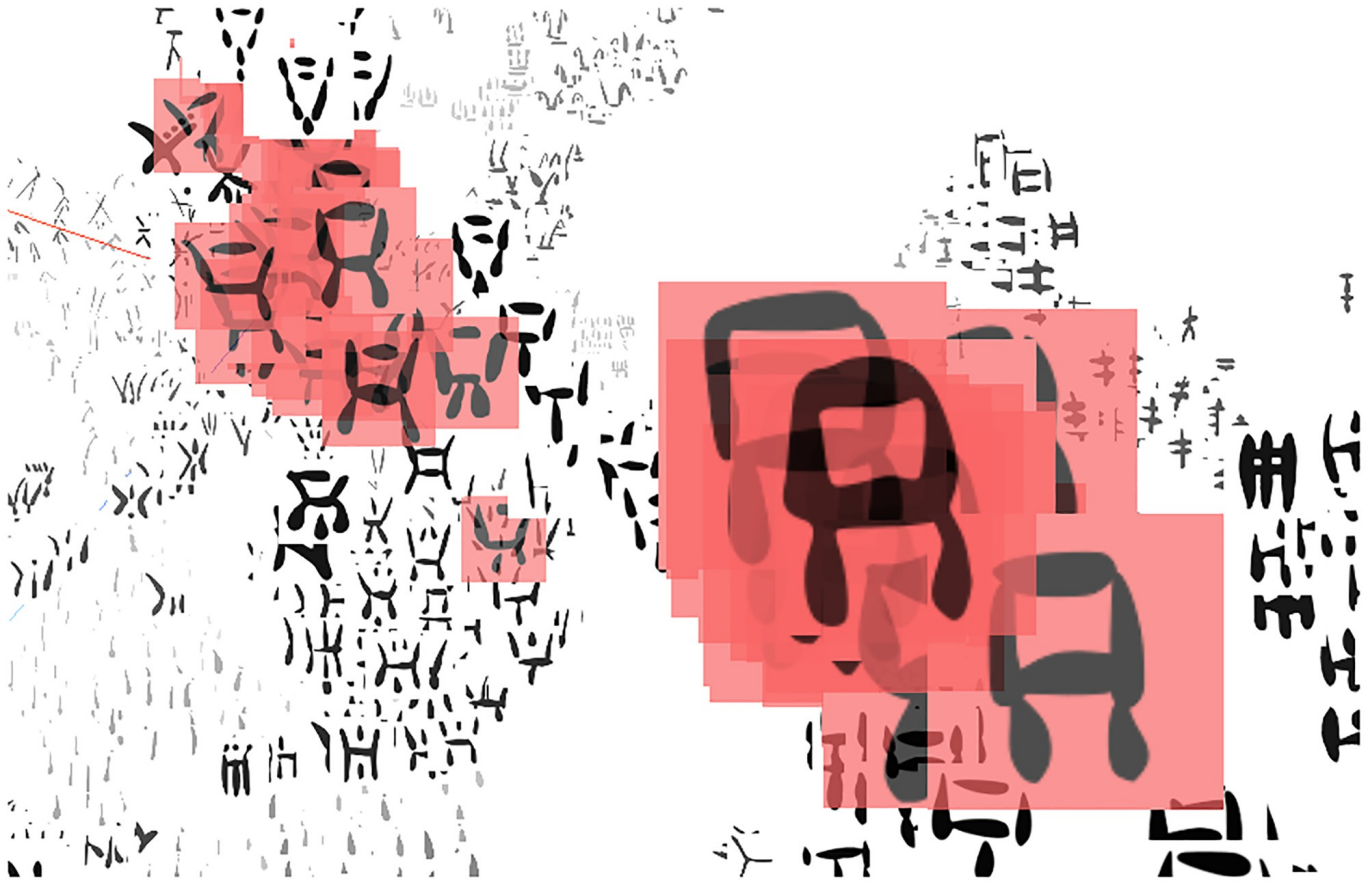
Separation of a CM grapheme in two groups in the 3D scatter plot. Example of sign 097.

We produced a 2560-dimensional representation obtained by applying the proposed neural model, namely *Sign2Vec_d_*, and the baseline, DeepClusterv2 (hosted at http://corpora.ficlit.unibo.it/INSCRIBE/PaperCM/). In the resulting 3D scatter plot, we observed that at the macro-level CM signs were distributed in two main groups: at its periphery, the hypersphere contained mainly signs from clay tablets, whereas at the core we found mostly signs inscribed on other types of objects (Fig 6). This separation is similar to the traditional divisions of CM1 and CM2, though not completely equivalent, as signs from inscriptions classed as CM3 are found both on clay tablets and other media. At closer range, we observed the same tendency: some of the consensually established CM graphemes appear either in a single “filament” (meaning that the sign images stay on the same branch of the plot) or as two clusters, but along an invisible axis traced from the core to the periphery. Thus, the instances of these graphemes inscribed on clay tablets (i.e., CM2 and some CM3 inscriptions) appear mainly at the outskirts of the plot, whereas attestations on other kinds of media (i.e., CM1 and other CM3 inscriptions) are placed mainly at the center (see e.g., sign 097 in Fig 7).

Importantly, while filaments or continua of clusters appear to represent graphemes (at least some), single clusters often do not. In fact, clusters in different parts of the scatter plot often blended sign shapes that scholars have classed as distinct graphemes (for example, because they co-exist and contrast in the same inscription).

Hence, we inferred that the model tended to arrange individual graphemes as filaments of sign images running from the core to the peripheral parts of the sphere, whereas the different layers of said sphere mainly reflect specific paleographic styles of signs. Because the outermost layer mainly displays signs from clay tablets (classed as CM2 as well as CM3), which characteristically are more angular (“squarish”) shapes and have fewer strokes, we also inferred that this layer reflects such shapes for multiple graphemes. That our model arranged CM signs in this way without supervision provides independent evidence in favor of the hypothesis that CM2 is not a distinct writing system, but rather a specific script style employed on clay tablets from Enkomi. If this is correct, the implication is that certain sign shapes peculiar to CM2 are not separate graphemes that are not part of the script found in CM1 and CM3 inscriptions. Rather, they would constitute variants of signs found on the other subcorpora, but with different forms (generally more angular or ‘compressed’, and/or with less strokes). Another implication of the model is that we should expect to find certain shapes to be arranged along the same core-periphery axis if they were variants of the same CM grapheme. We therefore used this property of the model to test the hypothesis that certain signs individuated by Olivier are rather allographs. However, before doing so, we need to consider one potential issue with the data.

### Preliminary relabelling of single signs

**Fig 8:**
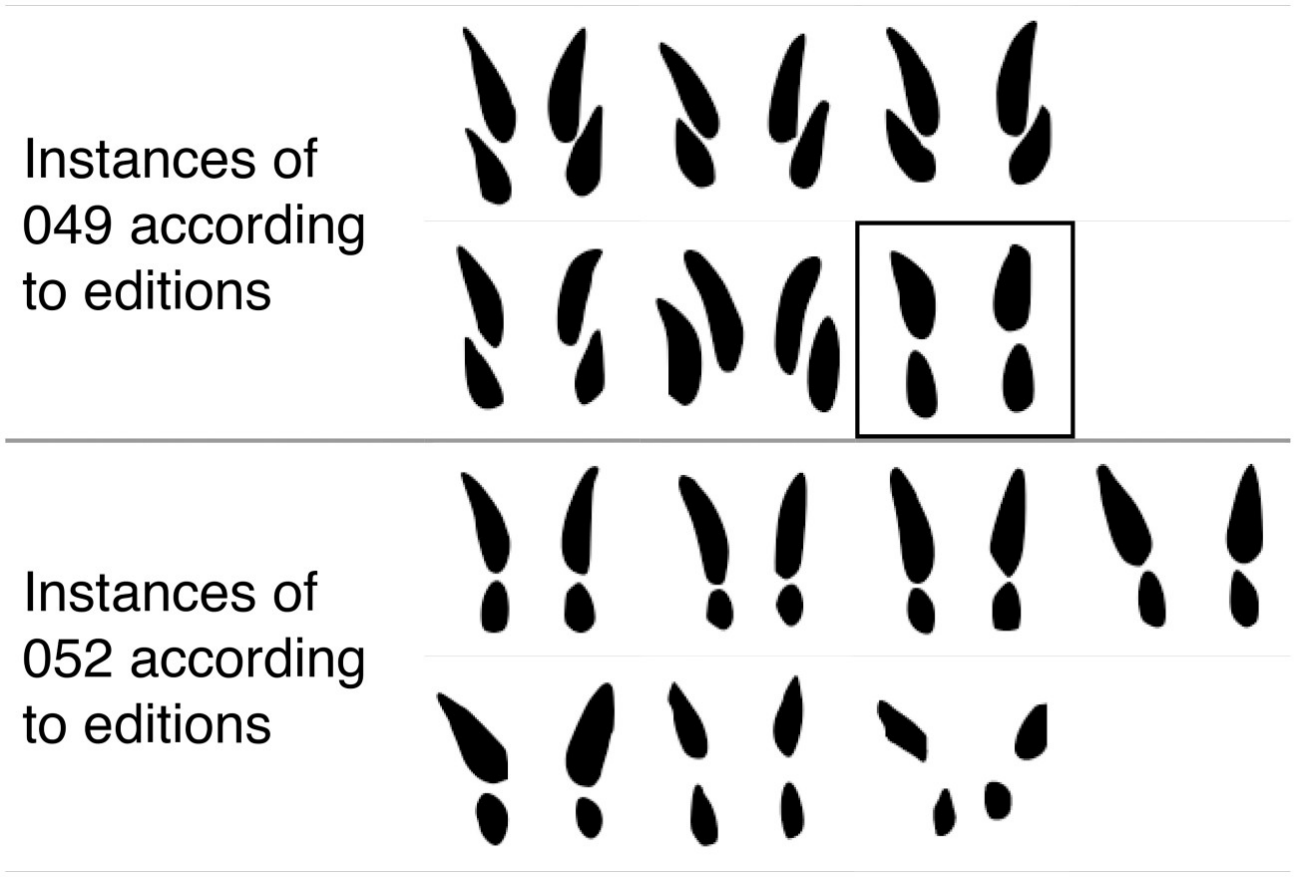
Example of sign with incoherent label (reading) in the editions of CM [5]: The sign marked with a contour has been transcribed as 049, even though its shape is consistent with 052. Such cases were tested for correction by means of a non-parametric Mann–Whitney U-test.

The 3D scatter plot obtained from a t-SNE projection of the 2560-dimensional concatenation of the 20 models outputs highlighted 27 instances of signs whose reading (transcription) in the published editions is incoherent even in terms of the conventional classification (example in Fig 8, and for which corrections have previously been proposed [3] in most cases. In other words, some signs appear clustered with similar or identical shapes in the scatter plot, but their labels (which follow the reference transcriptions) differ. These problematic labels include cases that are just obvious misprints in Olivier’s edited corpus [5]. As subsequent quantitative analyses of the results were based also on these labels, any incorrect transcription would affect their accuracy. Thus, we performed a set of preliminary tests on both DeepClusterv2 and *Sign2Vec_d_* to validate suggested corrections for these 27 instances (S3 and S4 Tables respectively).

We used the cosine distances between sign images to test statistically whether a proposed correction was more supported by the models than the “conventional” reading. Notice that the cosine distance is a valid metric for the vector space, as it is the distance metric used by the K-Means step of DeepCluster-v2. For each sign with a problematic transcription, we applied a non-parametric Mann-Whitney U-test [34] comparing the distances between the sign in question and the transcription vs. the distances between the sign and the proposed correction across all 20 models. Instead of using the concatenated, 2560-dimensional vector representation of signs, we derived the distances between the signs in all 20 vector spaces and compared their distribution by means of the statistical test. We set the significance value at 0.05 and applied corrections where the null hypothesis held, i.e., when the population of distances from the sign to the correction was smaller than the population of distances from the sign to the current reading. We applied this method to both the DeepClusterv2 and *Sign2Vec_d_* models. Out of a total of 31 tests performed, the results led us to re-label 26 and 20 single signs, respectively.

### The paleographic vector

We divided our dataset in two subgroups, reflecting the core/periphery separation displayed by the 3D scatter plot rather than the tripartite division into CM1, CM2 and CM3. Thus, one subset was termed *Tablet* and comprised inscriptions on clay tablets (again, the equivalent of CM2 and part of CM3); the other was labelled *Other* and, as the name implies, it included all other documents (CM1 and another part of CM3).

**Fig 9:**
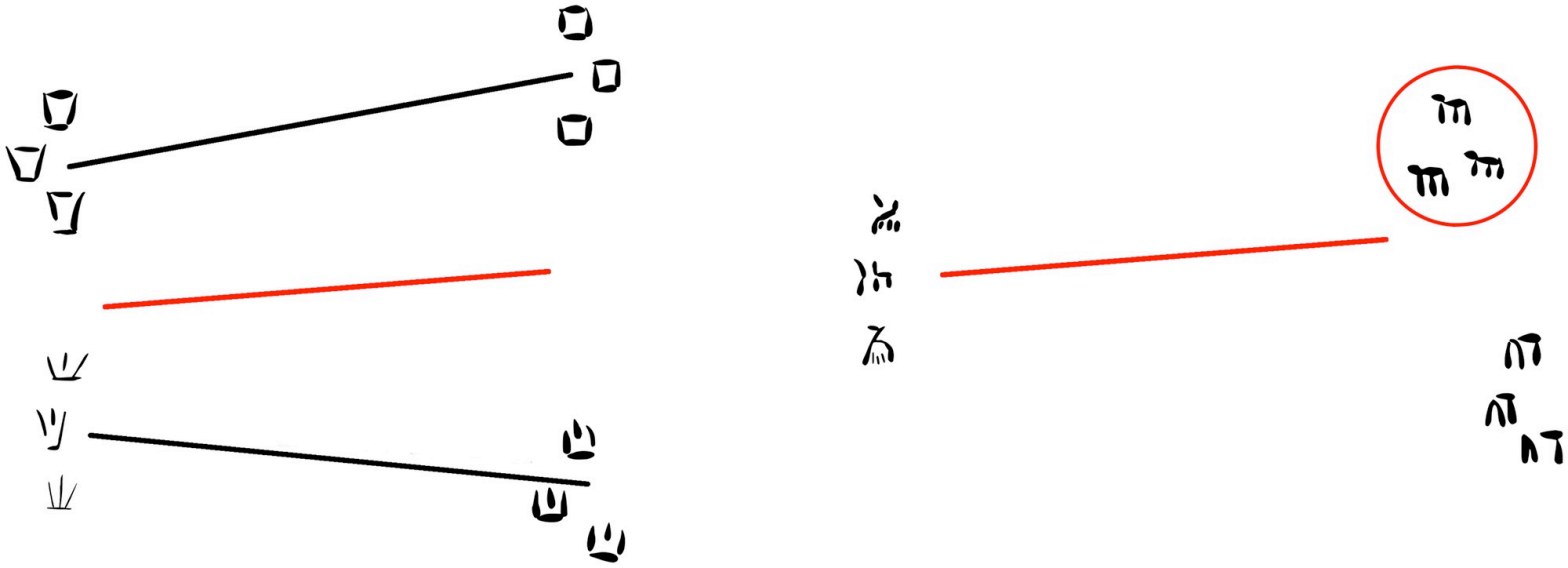
On the left: The paleographic vector (red) was first calculated from the difference vector (black) between centroids of signs from clay tablets and centroids of signs from other documents. On the right: the vector was applied to find a missing correspondence between signs shapes from the same two sets.

We then hypothesized that in the 2560 dimensional space, obtained by concatenating the latent representation of signs from the 20 models, we could find a vector that would encode the arrangements of instances of a single grapheme along this *Other*-*Tablet* axis. First and foremost, we would expect this to be true of signs which scholars already agree are well-attested and shared by the two larger subcorpora of CM, CM1 and CM2 (which means both clay tablets and other types of inscriptions). Thus, if the vector existed, it could then be used to also find potentially missing correspondences between signs in the *Other* subset and their allographs in the *Tablet* subset (see Fig 9). Put differently, we would obtain evidence that some signs so far catalogued separately are allographs if the vector encoded the same *Other* >*Tablet* direction for sign shapes accepted as single graphemes, and sign shapes thought to represent distinct graphemes (according to Olivier’s classification).

Formally, the paleographic vector *v*_*p*_ is defined as follows:

$$\begin{array}{c} v_{p}=\frac{1}{N}\sum_{i=1}^{N}[c\left(T_{i}\right)-c\left(O_{i}\right)] \end{array}$$ (1)

$$\begin{array}{c} c\left(X\right)=\frac{1}{\left|X\right|}\sum_{x\in X}x \end{array}$$ (2)

Where:

- N is the total number of signs present on both types of documents;
- *T*_*i*_, *O*_*i*_ are sets containing the attestations of sign i that are on tablets (*Tablet*) and on other documents (*Other*), respectively;
- *c*(*X*) denotes the centroid (mean) of a given set.

In Eq 1, we use the centroids of signs in the two subgroups to compute an average direction for the alignment of sign variants from clay tablets (*Tablet*) and sign variants from other types of inscription (*Other*).

If this paleographic vector, *v*_*p*_, is able to encode this direction, we would expect that:

$$\begin{array}{c} \underset{i\in [1,|T|]}{argmin}\;\delta \left[c\left(O_{j}\right)+v_{p},c\left(T_{i}\right)\right]=i^{\prime }\;\;\;\Rightarrow \;\;\;O_{j}\approx T_{i^{\prime }} \end{array}$$ (3)

Where *T* is the set of all sets of signs on tablets and *δ* is our distance metric, the cosine distance. We use ≈ to denote that two sets contain the same sign. We applied the vector to the centroid of the attestations of a given sign as found on inscriptions that are not clay tablets and looked for the closest centroid among the signs from tablets. We then expect *T*_*i*′_ and *O*_*j*_ to be the same grapheme.

### Validating the paleographic vector

**Fig 10:**
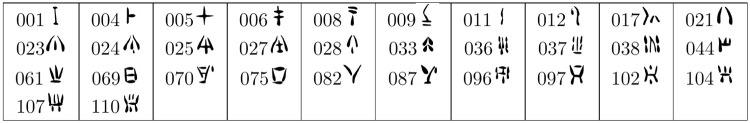
32 consensual signs attested both in the *Other* and *Tablets* subsets.

To test the hypothesis, we applied the paleographic vector to 32 consensual graphemes attested in the two largest traditional subgroups, CM1 and CM2 (always insofar as they are also present in our dataset): 001, 004, 005, 006, 008, 009, 011, 012, 017, 021, 023, 024, 025, 027, 028, 033, 036, 037, 038, 044, 061, 068, 070, 075, 082, 087, 096, 097, 102, 104, 107, 110 (Fig 10). The premise is the following: if the vector is largely able to match the variants of graphemes from CM1 with their counterparts in CM2, along the *Other*-*Tablet* axis, then it is valid. Notice that we considered CM signs attested in inscriptions classed as CM1 and CM2 even if they are not also attested in CM3 as well, because the latter subgroup is more limited in its number of inscriptions. Thus, if a sign is not yet attested in CM3 there is more chance that that absence is accidental.

We applied the vector to these 32 signs, taking as starting point their instances in the subset *Other*. After “projecting” it, for each sign we printed two rankings of the closest matches from the subset *Tablet*. These rankings (S5 and S6 Tables) were obtained form DeepClusterv2 and *Sign2Vec_d_*, respectively. The procedure meant at the same time the calculation of the vector and its validation: each time we applied the vector to a sign, we calculated it by excluding the sign under scrutiny from the computation of the vector. We wanted to test whether the *Other* >*Tablet* direction learned from the other 31 signs was sufficiently general to predict the direction of the excluded sign. The second aim was to compare the results of DeepClusterv2 and *Sign2Vec_d_* and determine whether the context-aware version of the model effectively achieved more accurate results. The results (Table 3) imply that the paleographic vector accurately reconstructs the match between Other and Tablet instances of the same grapheme and does it more accurately in its context aware *Sign2Vec_d_* version. We evaluate performance by using top-N accuracy, which measures the amount of tests in which the expected sign is found in the top N positions when the paleographic vector is applied. *Sign2Vec_d_* has a top-one accuracy of 0.69 while its top-two accuracy is 0.81. This means that for 22 out of 32 signs (≈69%) the correct *Tablet* instances were the first (closest) prediction, but if we also considered the signs that appear as second-closest prediction, the percentage of success raises to 81%.

**Table 3:** Application of the paleographic vector to consensual graphemes using both DeepClusterv2 and *Sign2Vec_d_*.

Model	Top-1 Accuracy	Top-2 Accuracy	Top-3 Accuracy	Top-5 accuracy
DeepClusterv2	0.66	0.75	0.81	0.97
Sign2Vec_d_	**0.69**	**0.81**	**0.94**	**1.0**

To assess the probability that this highly accurate result is the product of chance, we performed a simple test using a binomial distribution. We used the top-one accuracy value in DeepClusterv2, as it is the lowest level of accuracy attained by one of our models (Table 3). We needed to use:

P(X≥c)=∑k=cn(nk)pk(1-p)n-kX∼B(n,p) (4)

Where:

- *c* = 21 is the number signs correctly found in first position from DeepClusterv2;
- *n* = 32 is the number of tests that we performed;
-
$p=\frac{1}{64}$ is the probability of randomly finding the correct sign from 64 alternatives.

The formula shows that the probability of obtaining 21 or more correct answers is 1.28*10^−30^, which shows just how improbable it is that these results are the product of chance. Additionally, we can compute the expected value of the distribution, which is given by $n*p=32*\frac{1}{64}$. In other words, if we matched the signs randomly, we would predict a single sign correctly only half of the time. These results demonstrate the overall validity of the paleographic vector and the greater accuracy of the *Sign2Vec_d_* model.

### The vector as evidence of allography

A set of hypotheses has been put forward [3] which question the validity of the tripartite division and imply that several pairs of sign shapes inventoried separately in Olivier are rather variants of the same grapheme. We tested whether the paleographic vector supports these hypotheses, focusing on two types of situations:

- **Type 1**: Hypotheses of complementary distribution: these propose the merger of pairs of signs where one is allegedly exclusive to CM1 and the other is supposedly peculiar to CM2;
- **Type 2**: Hypotheses that involve the merger of two signs, one scarcely attested and present only in the CM1 subcorpus, the other present in all subcorpora.

**Fig 11:**
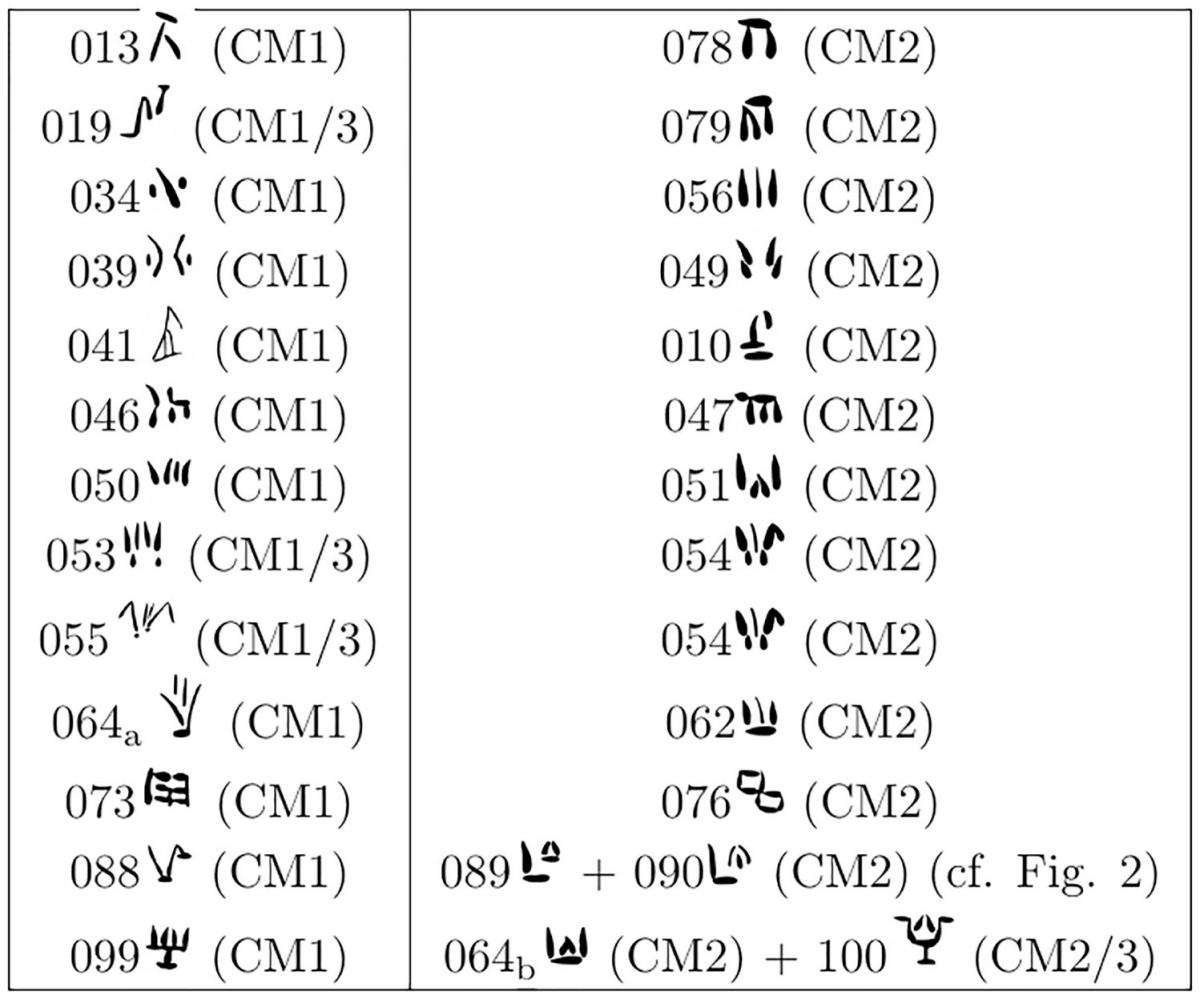
Pairs of CM1 (left column) / CM2 (right column) signs in complementary distribution and hypothesized as variants of the same grapheme.

In both types of hypotheses, we sought a correspondence that is potentially missing between a sign shape restricted to the subgroup *Other* and its possible *Tablet* counterpart. By applying the vector to a sign restricted to *Other* (taken as point of departure) and projecting it towards the peripheral layer of the hypersphere, we obtained the closest matches in *Tablet* (excluding the sign of departure from the rankings with the calculated distances). Thus, for example, Valério [3, 4] hypothesizes that 039 (supposedly restricted to CM1) is merely the allograph of 049 (allegedly an innovation of CM2). To test this proposal, we want to see if the vector indicated 049 as the closest *Tablet* match for 039, which in the scatter plot lied in the subset *Other*. We will now focus on the first type of proposal, involving signs in complementary distribution. The tests of Type 1 (hypotheses of complementary distribution) we performed involve the pairs listed in Fig 11. All these potential pairs of allographs have been proposed in [3, 4], and we tested also the more tentative suggestion for a merger of the pair 073 (CM1/3) + 076 (CM2). These hypotheses were based on complementary distributions between two very similar sign shapes, one supposedly attested only CM2 and another allegedly present only in CM1 or both in CM1 and CM3 (including CM3 clay tablets). This kind of distribution is not directly matched by the *Other* / *Tablet* separation observed in the scatter plot, which reveals a broad distinction between signs from clay tablets and signs from other supports. However, this by no means needs to be taken as an indication that allographs of a sign need to be identical on all clay tablets, both at Ugarit (= CM3) and Enkomi (= CM2). In other words, we contend that we can test the notion that the equivalent of 073 on the clay tablets from Enkomi is 076, while the clay tablets from Ugarit still employed the same shape as other media (073). Other hypotheses involve details that need to be accounted for:

- It has been hypothesized [3] that 053 (attested in CM1 and CM3), 054 (CM2) and 055 (CM3) are the same grapheme; this had two be tested separately, by applying the vectors to the pairs 053–054 and 055–054.
- What Olivier classed as sign 064 and deemed as attested both in CM1 and CM2, was originally catalogued as two different signs by Masson [1]: 064 (CM1) and 065 CM2). It has now been argued [3, 4] based on the different positional distributions of these shapes that indeed two different graphemes are represented: the 064 of CM1 (henceforth 064_a_), mainly sequence-initial, was suggested as the counterpart of the 062 of CM2; and the 064 of CM2 (henceforth 064_b_), mostly sequence-final, was hypothesized as the counterpart of sign shape 099 of CM1/3 (in addition to shape 100 from CM3). We tested these two parallel mergers.
- 089 and 090 (CM1) have been hypothesized as representing the same sign and therefore the joint counterpart of 088 (CM2). Thus, we tested whether both were indicated as the closest matches.

We tested the 13 hypotheses in Fig 11 by projecting the vector from the shapes attested in CM1 (013, 019, 034, 039, 041, 046, 050, 053, 055, 064a, 073, 088, 099). The acceptable targets were all sign shapes restricted to the subset *Tablet* in our dataset (010, 029, 040, 047, 049, 051, 054, 056, 060, 062, 064_b_, 074, 076, 078, 079, 080, 089, 090, 095, 100). The vector yielded the expected result with 8/13 top-one accuracy and 9/13 top-two accuracy (S7 Table). As with the validation process, we estimated the probability that our results were the product of chance. As we have two tests (088 vs. 089/090, 099 vs 064_b_/100) that sought a match with two target *Tablet* signs, we needed to consider multiple success probabilities, so we resorted to the Poisson binomial distribution. The probability of having 8 or more correct matches by chance was estimated to be very low (p-value = 1.0*10^−7^). We also calculated the mean number of successes that such a distribution would obtain, by summing the probabilities of each successful event:

$$\begin{array}{c} \sum_{i=0}^{n}p_{i}=\frac{15}{20} \end{array}$$ (5)

Therefore, if we chose the matching signs randomly, we would on average obtain less than one of the matches right ($\frac{15}{20}=0.75$). Thus, the probability of achieving the above results by chance is very low, and we interpret them as strong independent evidence in support of the allography hypotheses.

**Fig 12:**
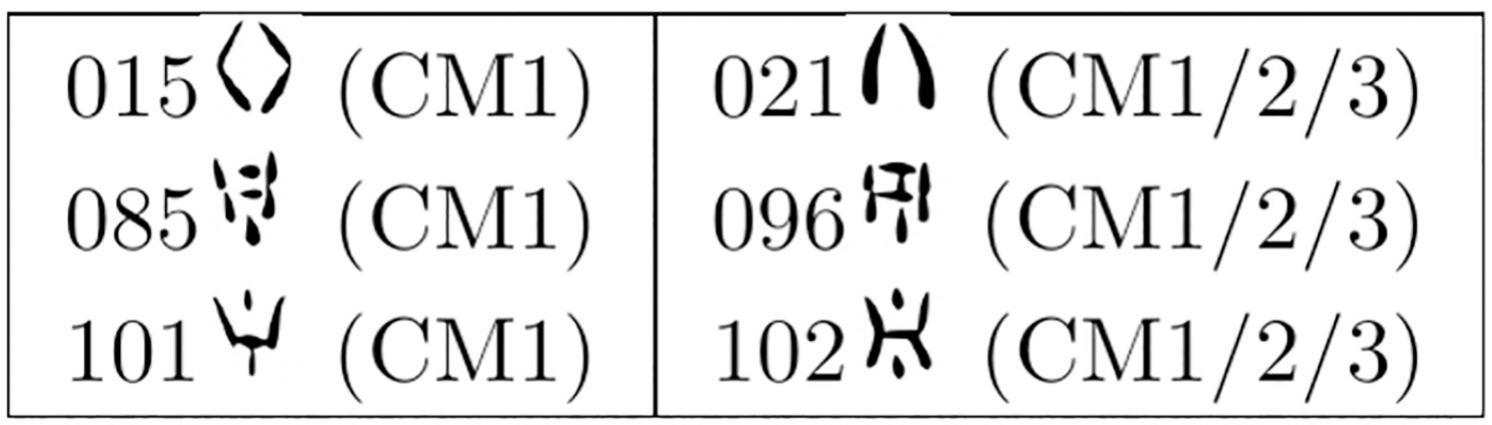
Pairs comprised of a shape attested only in CM1 (left column) and a shape attested in all sub-corpora (right column), hypothesized as variants of the same grapheme [3].

We also tested three hypotheses of Type 2. In this case, both the starting and target sign shapes are present (as sign images) at the core of the scatter plot (*Other*), but we still expect the vector to direct us from there to the correct target at the periphery (*Tablet*). Thus, we tested 015, 085 and 101 (attested only in CM1) as potential allographs of the better attested 021, 096, and 102 (CM1–2-3), respectively (Fig 12). The result was again largely positive: 2/3 top-one and top-two accuracy (S8 Table). We then applied the Poisson binomial distribution and obtained a p-value of 1.7*10^−3^. Moreover, the mean value of the distribution is 0.07, implying that we would find a correct match by chance less than 1 out of 10 times.

Altogether, these results show that the vector aligned instances of consensual graphemes (69% at top one) and signs separated by Olivier (11 out of 16 tests, or 68.75%) with a nearly identical rate of success.

## Conclusions

The vectors obtained from our *Sign2Vec_d_* unsupervised deep-learning model largely separated CM signs found on clay tablets (periphery) from signs attested on other supports (core), as observed in the resulting 3D scatter plot. Moreover, while following this separation, the variants of several consensual graphemes as found on clay tablets and other media were still aligned along an invisible axis running from the core to the periphery of the scatter plot. This is consistent with the notion that the main differentiation among CM signs concerns how the paleographic style of signs written on tablets diverges from that of signs on other types of inscriptions. By contrast, nothing in the model is consistent with a separation of signs traditionally seen as peculiar to one subcorpus (CM1, CM2, or CM3) and diagnostic of distinct CM writing systems. A pattern of the latter kind is what might have suggested structural divergences (presence or absence of graphemes) rather than stylistic variation.

A priori, it was questionable to what extent the model’s representation of the relationships between CM signs was consistent with the reality of the script (or scripts) present in the corpus. To validate it, we took as ground truth 32 CM signs whose entity as graphemes is not questioned by either proponents or opponents of the tripartite division of CM as maintained in the work of Olivier (because they are well-attested and show relatively little graphic variation). That 69% of these graphemes are arranged along a core-periphery vector that reflects the stylistic differentiation of signs inscribed on clay tablets (and is mathematically regular) strongly implies that the model’s representation is grounded. Moreover, statistical tests show that it is highly improbable that this result is accidental.

The traditional classification that divides CM in three alleged scripts largely rests on the assumption that significant numbers of signs exist which are peculiar, and innovations within a given subgroup. The model reported here provides measurable evidence against this assumption, suggesting that signs supposedly diagnostic of different scripts are incorrectly identified. For example, the paleographic vector supports the notion that certain pairs of sign shapes hitherto treated as separate entities (e.g. 088 and 089+090), and potentially peculiar to a given subcorpus of CM (CM1 in the case of 088, CM2 in the case of 089 and 090), are allographs. This is because these pairs of shapes are arranged in the same way mere variants of consensual independent graphemes are (e.g., 087 as attested on clay tablets and other media). Thus, if the latter have been accepted as single entities, we suggest that by reasons of coherence the former should also be considered as potential graphemes. With these results, the tripartite division loses substantial empirical basis, and the hypothesis that it is invalid (as previously put forward based on paleographic and distributional evidence) gains strength.

The implications tied to Masson’s 1974 starting assumption [1] that different languages were present in the corpus of CM inscriptions cannot be tested in the scope of this paper nor we can infer anything new on the language(s) represented based on our results.

If all hypotheses of allography were correct, the failure of 31.25% of the vector tests would likely not be tied to issues of sampling. The sign shapes involved in the unsupported hypotheses were not, as a rule, less attested than shapes whose merger was supported. Thus, the imprecise matches in pairs such as 013–078 or 019–079 may have to do with variations in shape or distribution beyond the average variability found among the consensual signs that yielded the paleographic vector. That these sign shapes differ more clearly reflects the lack of consensus on their classification.

Overall, the application of an unsupervised approach to CM has shown that neural networks can be fruitfully applied to ancient undeciphered scripts. In particular, the application of *Sign2Vec_d_* provided an independent way to test hypotheses of sign and script classification. Nevertheless, it remains the case that any application of neural networks to undeciphered scripts must be developed *ad hoc*, as no general method is single-handedly effective in reaching solid and cogent results. In the future, other aspects of CM should be investigated, such as its relationship with the undeciphered syllabic Linear A script from Minoan Crete and the script historically developed from it on Cyprus, the 1st-millennium BCE Cypro-Greek syllabary.

## Supporting information

S1 Table List of CM inscriptions included in and excluded from the dataset. NB: Reference edition refers to the source of the drawing used in the text and is not necessarily the first publication of the inscribed object.
(PDF)

S2 Table DeepClusterv2 hyperparameters. (PDF)

S3 Table Preliminary corrections to incoherent sign labels using non-parametric Mann-Whitney U test on the outputs of DeepClusterv2. Since both tests for 086 and 112 (inscription ##211) were positive, we performed an additional test that compared them. We applied 112 as a correction even if the statistical test was inconclusive, since 112 was favored by the model.
(PDF)

S4 Table Preliminary corrections to incoherent sign labels using non-parametric Mann-Whitney U test on the outputs of Sign2Vec_d_. Since both tests for 086 and 112 (inscription ##211) were positive, we performed an additional test that compared them. The same is true for 102 and 024 (inscription ##215).
(PDF)

S5 Table Validation of CM *Other* > *Tablet* paleographic vector for DeepClusterv2. The correct targets are marked in bold.
(PDF)

S6 Table Validation of CM *Other* > *Tablet* paleographic vector for Sign2Vec_d_. The correct targets are marked in bold.
(PDF)

S7 Table Test of hypothesized mergers of signs in complementary distribution. The hypothesized correct targets are marked in bold. Some matches are impossible, because the starting *Other* sign shape and the target *Tablet* one are known to coexist in an inscription, where they are contrastive graphemes. Thus, 055 contrasts with both 051 and 095 on clay tablet ##215. 073, 074 and 095 are also contrastive on the same inscription. Impossible matches are discarded and stricken through in the table.
(PDF)

S8 Table Test of hypothesized mergers of sign shapes attested only in CM1 with sign shapes attested in all subcorpora. The hypothesized correct targets are marked in bold. Impossible matches are discarded and stricken through in the table.
(PDF)
