# Supplementary material for: Unsupervised deep learning supports reclassification of Bronze age cypriot writing system
Source: PLoS One. 2022 Jul 14;17(7):e0269544. doi: 10.1371/journal.pone.0269544 (PMC9282481; doi:10.1371/journal.pone.0269544)
Supplement: S1 Table — NB: Reference edition refers to the source of the drawing used in the text and is not necessarily the first publication of the inscribed object. (PDF) [file pone.0269544.s001.pdf]

Inscription	Reference edition	Included	Reason for exclusion
##001. ENKO Atab 001	Olivier (2007)	NO	Isolated CM0 sub-corpus
##002. ENKO Abou 001	Olivier (2007)	YES	
##003. ENKO Abou 002	Olivier (2007)	YES	
##004. ENKO Abou 003	Olivier (2007)	YES	
##005. ENKO Abou 004	Olivier (2007)	YES	
##006. ENKO Abou 005	Olivier (2007)	YES	
##007. ENKO Abou 006	Olivier (2007)	YES	
##008. ENKO Abou 007	Olivier (2007)	YES	
##009. ENKO Abou 008	Olivier (2007)	NO	All signs damaged / no drawing
##010. ENKO Abou 009	Olivier (2007)	NO	All signs damaged
##011. ENKO Abou 010	Olivier (2007)	YES	
##012. ENKO Abou 011	Olivier (2007)	YES	
##013. ENKO Abou 012	Olivier (2007)	YES	
##014. ENKO Abou 013	Olivier (2007)	YES	
##015. ENKO Abou 014	Olivier (2007)	YES	
##016. ENKO Abou 015	Olivier (2007)	YES	
##017. ENKO Abou 015bis	Olivier (2007)	NO	All signs damaged / no drawing
##018. ENKO Abou 016	Olivier (2007)	YES	
##019. ENKO Abou 016bis*	Olivier (2007)	NO	Unepigraphic
##020. ENKO Abou 017	Olivier (2007)	YES	
##021. ENKO Abou 018	Olivier (2007)	YES	
##022. ENKO Abou 019	Olivier (2007)	YES	
##023. ENKO Abou 020	Olivier (2007)	YES	
##024. ENKO Abou 021	Olivier (2007)	YES	
##025. ENKO Abou 022	Olivier (2007)	YES	
##026. ENKO Abou 023	Olivier (2007)	YES	
##027. ENKO Abou 024	Olivier (2007)	YES	
##028. ENKO Abou 025	Olivier (2007)	YES	
##029. ENKO Abou 026	Olivier (2007)	YES	
##030. ENKO Abou 027	Olivier (2007)	YES	
##031. ENKO Abou 028	Olivier (2007)	NO	All signs damaged
##032. ENKO Abou 029	Olivier (2007)	YES	
##033. ENKO Abou 030	Olivier (2007)	YES	
##034. ENKO Abou 031	Olivier (2007)	YES	
##035. ENKO Abou 032	Olivier (2007)	YES	
##036. ENKO Abou 033	Olivier (2007)	YES	
##037. ENKO Abou 034	Olivier (2007)	YES	
##038. ENKO Abou 035	Olivier (2007)	YES	
##039. ENKO Abou 036	Olivier (2007)	YES	
##040. ENKO Abou 037	Olivier (2007)	YES	
##041. ENKO Abou 038	Olivier (2007)	YES	
##042. ENKO Abou 039	Olivier (2007)	YES	
##043. ENKO Abou 040	Olivier (2007)	YES	
##044. ENKO Abou 041	Olivier (2007)	YES	
##045. ENKO Abou 042	Olivier (2007)	YES	
##046. ENKO Abou 043	Olivier (2007)	YES	
##047. ENKO Abou 044	Olivier (2007)	YES	
##048. ENKO Abou 045	Olivier (2007)	YES	
##049. ENKO Abou 046	Olivier (2007)	YES	
##050. ENKO Abou 047	Olivier (2007)	YES	
##051. ENKO Abou 048	Olivier (2007)	YES	
##052. ENKO Abou 049	Olivier (2007)	YES	

Inscription	Reference edition	Included	Reason for exclusion
##053. ENKO Abou 050	Olivier (2007)	YES	
##054. ENKO Abou 051	Olivier (2007)	YES	
##055. ENKO Abou 052	Olivier (2007)	YES	
##056. ENKO Abou 053	Olivier (2007)	YES	
##057. ENKO Abou 054	Olivier (2007)	YES	
##058. ENKO Abou 055	Olivier (2007)	YES	
##059. ENKO Abou 056	Olivier (2007)	YES	
##060. ENKO Abou 057	Olivier (2007)	YES	
##061. ENKO Abou 058	Olivier (2007)	YES	
##062. ENKO Abou 059	Olivier (2007)	NO	All signs damaged
##063. ENKO Abou 060	Olivier (2007)	YES	
##064. ENKO Abou 061	Olivier (2007)	YES	
##065. ENKO Abou 062	Olivier (2007)	YES	
##066. ENKO Abou 063	Olivier (2007)	YES	
##067. ENKO Abou 064	Olivier (2007)	YES	
##068. ENKO Abou 065	Olivier (2007)	YES	
##069. ENKO Abou 066	Olivier (2007)	YES	
##070. ENKO Abou 067	Olivier (2007)	YES	
##071. ENKO Abou 068	Olivier (2007)	YES	
##072. ENKO Abou 069	Olivier (2007)	YES	
##073. ENKO Abou 069ter*	Olivier (2007)	NO	Unepigraphic
##074. ENKO Abou 070	Olivier (2007)	NO	All signs damaged / no drawing
##075. ENKO Abou 071	Olivier (2007)	YES	
##076. ENKO Abou 072	Olivier (2007)	YES	
##077. ENKO Abou 073	Olivier (2007)	YES	
##078. ENKO Abou 074	Olivier (2007)	YES	
##079. ENKO Abou 075	Olivier (2007)	YES	
##080. ENKO Abou 076	Olivier (2007)	YES	
##081. ENKO Abou 077	Olivier (2007)	NO	Missing / drawing not verified
##082. ENKO Abou 078	Olivier (2007)	YES	
##083. ENKO Abou 079	Olivier (2007)	YES	
##084. ENKO Abou 080	Olivier (2007)	YES	
##085. ENKO Abou 081	Olivier (2007)	YES	
##086. ENKO Abou 082	Olivier (2007)	YES	
##087. ENKO Abou 083	Olivier (2007)	YES	
##088. HALA Abou 001	Olivier (2007)	YES	
##089. HALA Abou 002	Olivier (2007)	YES	
##090. KITI Abou 001	Olivier (2007)	YES	
##091. KITI Abou 002	Olivier (2007)	YES	
##092. ATHI Adis 001	Olivier (2007)	NO	Cypro-Greek inscription
##093. ENKO Aost 001	Olivier (2007)	YES	
##094. ENKO Aost 002	Olivier (2007)	YES	
##095. ENKO Apes 001	Olivier (2007)	YES	
##096. ENKO Apla 001	Olivier (2007)	NO	All signs damaged
##097. ENKO Arou 001	Olivier (2007)	YES	
##098. KALA Arou 001	Olivier (2007)	YES	
##099. KALA Arou 002	Olivier (2007)	YES	
##100. KALA Arou 003	Olivier (2007)	YES	
##101. KALA Arou 004	Olivier (2007)	YES	
##102. KALA Arou 005	Olivier (2007)	YES	
##103. PSIL Asta 001	Olivier (2007)	YES	
##104. ALAS Avas 001	Olivier (2007)	YES	
##105. ARPE Avas 001	Olivier (2007)	YES	
##106. ATHI Avas 001	Olivier (2007)	YES	
##107. ATHI Avas 002	Olivier (2007)	NO	All signs damaged
##108. ENKO Avas 001	Olivier (2007)	YES	

Inscription	Reference edition	Included	Reason for exclusion
##109. ENKO Avas 002	Olivier (2007)	YES	
##110. ENKO Avas 003	Olivier (2007)	YES	
##111. ENKO Avas 004	Olivier (2007)	YES	
##112. ENKO Avas 005	Olivier (2007)	YES	
##113. ENKO Avas 006	Olivier (2007)	YES	
##114. ENKO Avas 007	Olivier (2007)	YES	
##115. ENKO Avas 008	Olivier (2007)	YES	
##116. ENKO Avas 009	Olivier (2007)	NO	All signs damaged
##117. ENKO Avas 010	Olivier (2007)	YES	
##118. ENKO Avas 011	Olivier (2007)	YES	
##119. ENKO Avas 012	Olivier (2007)	YES	
##120. ENKO Avas 013	Olivier (2007)	YES	
##121. ENKO Avas 014	Olivier (2007)	YES	
##122. HALA Avas 001	Olivier (2007)	YES	
##123. IDAL Avas 001	Olivier (2007)	YES	
##124. IDAL Avas 002	Olivier (2007)	NO	Classification and segmentation debated
##125. KALA Avas 001	Olivier (2007)	YES	
##126. KALA Avas 002	Olivier (2007)	YES	
##127. KATY Avas 001	Olivier (2007)	YES	
##128. KATY Avas 002	Olivier (2007)	YES	
##129. KATY Avas 003	Olivier (2007)	YES	
##130. KITI Avas 001	Olivier (2007)	YES	
##131. KITI Avas 002	Olivier (2007)	YES	
##132. KITI Avas 003	Olivier (2007)	YES	
##133. KITI Avas 004	Olivier (2007)	YES	
##134. KITI Avas 005	Olivier (2007)	YES	
##135. KITI Avas 006	Olivier (2007)	YES	
##136. KITI Avas 007	Olivier (2007)	NO	All signs damaged
##137. KITI Avas 008	Olivier (2007)	YES	
##138. KITI Avas 009	Olivier (2007)	YES	
##139. KITI Avas 010	Olivier (2007)	YES	
##140. KITI Avas 011	Olivier (2007)	YES	
##141. KITI Avas 012	Olivier (2007)	YES	
##142. KITI Avas 013	Olivier (2007)	YES	
##143. KITI Avas 014	Olivier (2007)	YES	
##144. KITI Avas 015	Olivier (2007)	YES	
##145. KITI Avas 016	Olivier (2007)	YES	
##146. KITI Avas 017	Olivier (2007)	YES	
##147. KITI Avas 018	Olivier (2007)	YES	
##148. KITI Avas 019	Olivier (2007)	YES	
##149. KOUR Avas 001	Olivier (2007)	YES	
##150. KOUR Avas 002	Olivier (2007)	NO	All signs damaged
##151. KOUR Avas 003	Olivier (2007)	YES	
##152. KOUR Avas 004	Olivier (2007)	YES	
##153. MAAP Avas 001	Olivier (2007)	YES	
##154. MAAP Avas 002	Olivier (2007)	YES	
##155. MAAP Avas 003	Olivier (2007)	YES	
##156. MAAP Avas 004	Olivier (2007)	YES	
##157. MARO Avas 001	Olivier (2007)	YES	
##158. MYRT Avas 001	Olivier (2007)	YES	
##159. MYRT Avas 002	Olivier (2007)	YES	
##160. TOUM Avas 001	Olivier (2007)	NO	No drawing
##161. KITI Iins 001	Olivier (2007)	YES	
##162. KITI Iins 002	Olivier (2007)	YES	
##163. KITI Ipla 001	Olivier (2007)	YES	
##164. ENKO Mbij 001	Olivier (2007)	NO	Quality of drawing insufficient

Inscription	Reference edition	Included	Reason for exclusion
##165. KALA Mbij 001	Olivier (2007)	YES	
##166. KALA Mbij 002	Olivier (2007)	YES	
##167. KITI Mexv 001	Olivier (2007)	YES	
##168. ENKO Mins 001	Olivier (2007)	YES	
##169. ENKO? Mins 002	Olivier (2007)	YES	
##170. PPAP Mins 001	Olivier (2007)	NO	Cypro-Greek inscription
##171. PPAP Mins 002	Olivier (2007)	NO	Cypro-Greek inscription(?)
##172. PPAP Mins 003	Olivier (2007)	NO	Cypro-Greek inscription(?)
##173. PYLA Mins 001	Olivier (2007)	YES	
##174. ENKO Mlin 001	Olivier (2007)	YES	
##175. ENKO Mlin 002	Olivier (2007)	YES	
##176. ENKO Mlin 003	Olivier (2007)	YES	
##177. PYLA Mlin 001	Olivier (2007)	YES	
##178. CYPR Mvas 001	Olivier (2007)	YES	
##179. CYPR Mvas 002	Olivier (2007)	YES	
##180. CYPR Mvas 003	Olivier (2007)	YES	
##181. CYPR Mvas 004	Olivier (2007)	YES	
##182. ENKO Mvas 001	Olivier (2007)	YES	
##183. ENKO Mvas 002	Olivier (2007)	YES	
##184. MYRT Mvas 001	Olivier (2007)	YES	
##185. MYRT Mvas 002	Olivier (2007)	YES	
##186. PPAP Mvas 001	Olivier (2007)	YES	
##187. ENKO Pblo 001	Olivier (2007)	YES	
##188. KITI Pblo 001	Olivier (2007)	YES	
##189. PPAP Pblo 001	Olivier (2007)	NO	Cypro-Greek inscription
##190. PPAP Pblo 002	Olivier (2007)	NO	Cypro-Greek inscription(?)
##191. KALA Ppla 001	Olivier (2007)	YES	
##192. KALA Ppla 002	Olivier (2007)	YES	
##193. CYPR? Psce 001	Olivier (2007)	NO	No drawing
##194. CYPR? Psce 002	Olivier (2007)	YES	
##195. CYPR? Psce 003	Olivier (2007)	YES	
##196. CYPR? Psce 004	Olivier (2007)	YES	
##197. CYPR? Psce 005	Olivier (2007)	YES	
##198. CYPR? Psce 006	Olivier (2007)	NO	No drawing
##199. ENKO Psce 001	Olivier (2007)	YES	
##200. ENKO? Psce 002	Olivier (2007)	YES	
##201. HALA Psce 001	Olivier (2007)	NO	No drawing
##202. KOUR Psce 001	Olivier (2007)	YES	
##203. PARA Psce 001	Olivier (2007)	YES	
##204. PYLA Psce 001	Olivier (2007)	YES	
##205. SALA Psce 001	Olivier (2007)	YES	
##206. PPAP Vsce 001	Olivier (2007)	NO	No drawing
##207. ENKO Atab 002a-b	Olivier (2007)	YES	
##208. ENKO Atab 003	Olivier (2007)	YES	
##209. ENKO Atab 004	Olivier (2007)	YES	
##210. RASH Aéti 001	Olivier (2007)	YES	
##211. RASH Aéti 002	Olivier (2007)	YES	
##212. RASH Atab 001	Olivier (2007)	YES	
##213. RASH Atab 002	Olivier (2007)	YES	
##214. RASH Atab 003	Olivier (2007)	YES	
##215. RASH Atab 004	Olivier (2007)	YES	
##216. RASH Mvas 001	Olivier (2007)	YES	
##217. SYRI Psce 001	Olivier (2007)	NO	Possible non-Cypriot sequence?
ADD##218. PARA Psce 002	Ferrara (2013)	YES	
ADD##219. APLI Psce 001*	Ferrara (2013)	NO	Single sign, status doubtful

Inscription	Reference edition	Included	Reason for exclusion
ADD##220. CYPR Psce 007	Ferrara (2013)	NO	All signs damaged / quality of drawing insufficient
ADD##221. DHEN Avas 001	Ferrara (2013)	NO	Not writing? (Potmarks?)
ADD##222. ENKO Apes 002*	Ferrara (2013)	NO	Single sign, status doubtful
ADD##223. ENKO Apes 003*	Ferrara (2013)	NO	Single sign, status doubtful
ADD##224. ENKO Pblo 002	Ferrara (2013)	YES	
ADD##225. ENKO Psce 003	Ferrara (2013)	NO	Not writing? (Status of signs debated)
ADD##226. ENKO Psce 004	Ferrara (2013)	NO	No drawing
ADD##227. ENKO Psce 005	Ferrara (2013)	YES	
ADD##228. ENKO Mins 003*	Ferrara (2013)	NO	Single sign / no drawing
ADD##229. ENKO Mins 004	Ferrara (2013)	YES	
ADD##230. ENKO Mins 005	Ferrara (2013)	NO	No drawing
ADD##231. KLAV Avas 001	Ferrara (2013)	YES	
ADD##232. IDAL Psce 001*	Ferrara (2013)	NO	Not writing
ADD##233. IDAL Avas 003	Ferrara (2013)	YES	
ADD##234. IDAL Pfus 001	Ferrara (2013)	NO	Missing / No drawing available
ADD##235. KALO Avas 001	Ferrara (2013)	NO	Quality of drawing insufficient
ADD##236. KITI Avas 020	Ferrara (2013)	NO	Single sign, status doubtful
ADD##237. KITI Avas 021	Ferrara (2013)	YES	
ADD##238. MAAP Avas 005	Ferrara (2013)	YES	
ADD##239. MARO Avas 002	Ferrara (2013)	YES	
ADD##240. MARO Avas 003	Ferrara (2013)	YES	
ADD##241. MARO Avas 004	Ferrara (2013)	YES	
ADD##242. SANI Avas 001	Ferrara (2013)	YES	
ADD##243. RASH Avas 001	Ferrara (2013)	NO	No drawing
ADD##244. TIRY Abou 001	Ferrara (2013)	YES	
ADD##245. TIRY Avas 001	Hirschfeld (1999: 72)	YES	
ADD##246. TIRY Avas 002	Davis, Maran and Wirghová (2014)	YES	
ADD##247. ENKO Abou 084	Valério (2014)	YES	
ADD##248. KOUR Avas 005	Valério (2014)	YES	
ADD##249. KOUR Avas 006	Valério (2014)	YES	
ADD##250. KOUR Avas 007	Valério (2014)	YES	
ADD##251. RASH Avas 002	Valério (2014)	NO	All signs damaged
ADD##252. CYPR? Psce 008	Valério (2014)	YES	
ADD##253. PPAP Psce 001	Valério (2014)	YES	
ADD##254. PPAP Mvas 002	Egetmeyer (2016)	NO	Drawing not available
Erimi-Kafkalla T.2/2	Hirschfeld (2012)	YES	
RS 1963	Valério (2016: 565)	YES	
Hishuley-Carmel bronze hoe	Valério and Davis (2017: 136)	YES	
