# Supplementary material for: Unsupervised deep learning supports reclassification of Bronze age cypriot writing system
Source: PLoS One. 2022 Jul 14;17(7):e0269544. doi: 10.1371/journal.pone.0269544 (PMC9282481; doi:10.1371/journal.pone.0269544)
Supplement: S2 Table — (PDF) [file pone.0269544.s002.pdf]

**Hyperparameter**	**Value**
Architecture	Resnet18
Base Learning Rate	4.8
Batch size	16;
Crops for assign	[0]
Epochs	100
Feature dimensions	128
Final learning rate	0.0048
Number iterations before prototypes freeze	300000
Hidden MLP size	2048
Max scale crops	[1.0, 0.6]
Min scale crops	[0.6, 0.4]
Number of crops	[6, 10]
Number of prototypes	[100,100,100]
Size of the crops	[80, 60]
Start warmup	0.3
Temperature	0.1
Warmup Epochs	10
Weight decay	1×10^{-6}
