# Supplementary material for: Unsupervised deep learning supports reclassification of Bronze age cypriot writing system
Source: PLoS One. 2022 Jul 14;17(7):e0269544. doi: 10.1371/journal.pone.0269544 (PMC9282481; doi:10.1371/journal.pone.0269544)
Supplement: S3 Table — Since both tests for 086 and 112 (inscription ##211) were positive, we performed an additional test that compared them. We applied 112 as a correction even if the statistical test was inconclusive, since 112 was favored by the model. (PDF) [file pone.0269544.s003.pdf]

Inscription	Sign Position	Published reading	Reference published reading	Proposed correction (Valério 2013, 2016)	Test Result	P-value
##011. ENKO Abou 010	r00.1	101	Olivier (2007)	102	Negative	1.0
##011. ENKO Abou 010	r00.3	013	Olivier (2007)	008	Positive	2.88×10^{-49}
##014. ENKO Abou 013	r00.1	075	Olivier (2007)	073	Positive	3.41×10^{-153}
##025. ENKO Abou 022	r00.3	008	Olivier (2007)	013	Positive	2.04×10^{-70}
##026. ENKO Abou 023	r00.2	075	Olivier (2007)	073	Positive	1.05×10^{-136}
##026. ENKO Abou 023	r00.5	075	Olivier (2007)	073	Positive	9.71×10^{-131}
##034. ENKO Abou 031	r00.2	095	Olivier (2007)	096	Positive	1.81×10^{-36}
##046. ENKO Abou 043	r00.2	068	Olivier (2007)	097	Positive	$4.33 \times 10^{-37}1$
##052. ENKO Abou 049	r00.2	068	Olivier (2007)	097	Positive	6.04×10^{-63}
##063. ENKO Abou 060	r00.2	087	Olivier (2007)	088	Positive	4.08×10^{-42}
##095. ENKO Apes 001	r00.7	064	Olivier (2007)	037	Negative	1.0
##098. KALA Arou 001	r15.4	070	Olivier (2007)	087	Positive	1.08×10^{-183}
##108. ENKO Avas 001	r00.2	006	Olivier (2007)	009	Positive	4.14×10^{-97}
##111. ENKO Avas 004	r00.4	008	Olivier (2007)	013	Positive	3.81×10^{-51}
##123. IDAL Avas 001	r00.3	068	Olivier (2007)	097	Positive	9.49×10^{-84}
##157. MARO Avas 001	r00.7	068	Olivier (2007)	097	Positive	1.35×10^{-69}
##179. CYPR Mvas 002	r00.4	008	Olivier (2007)	013	Positive	1.51×10^{-66}
##194. CYPR? Psce 002	r00.1	068	Olivier (2007)	097	Positive	6.60×10^{-5}
##207. ENKO Atab 002.B	r10003d.10	072	Olivier (2007)	070	Positive	1.13×10^{-2}
##207. ENKO Atab 002.B	r10004d.10	049	Olivier (2007)	052	Positive	5.39×10^{-13}
##208. ENKO Atab 003.A	r05d.1	005	Olivier (2007)	004	Positive	9.77×10^{-153}
##211. RASH Aéti 002	r00.3	064	Ferrara (2013)	086	Positive	2.47×10^{-25}
##211. RASH Aéti 002	r00.3	064	Ferrara (2013)	112	Positive	5.81×10^{-30}
##211. RASH Aéti 002	r00.3	086	N/A	112	Negative	0.08
##212. RASH Atab 002	r02.3	102	Olivier (2007)	104	Positive	3.57×10^{-59}
##215. RASH Atab 004.A	r08.1	103	Olivier (2007)	102	Negative	0.20
##215. RASH Atab 004.A	r08.1	103	Olivier (2007)	102	Positive	3.32×10^{-37}
##215. RASH Atab 004.A	r10.2	008	Olivier (2007)	013	Positive	-3.44×10^{-68}
##215. RASH Atab 004.B	r12.3	072	Olivier (2007)	073	Positive	1.15×10^{-2}
ADD##229. ENKO Mins 004	r00.2	027	Ferrara (2013)	025	Negative	1.0
ADD##233. IDAL Avas 003	r00.5	025	Ferrara (2013)	027	Negative	1.0
ADD##237. KITI Avas 021	r00.1	006	Ferrara (2013)	102	Negative	1.0
ADD##237. KITI Avas 021	r00.2	023	Ferrara (2013)	061	Positive	1.17×10^{-121}
ADD##242. SANI Avas 001	r00.2	082	Ferrara (2013)	053	Positive	4.48×10^{-61}
