# Supplementary material for: Unsupervised deep learning supports reclassification of Bronze age cypriot writing system
Source: PLoS One. 2022 Jul 14;17(7):e0269544. doi: 10.1371/journal.pone.0269544 (PMC9282481; doi:10.1371/journal.pone.0269544)
Supplement: S4 Table — Since both tests for 086 and 112 (inscription ##211) were positive, we performed an additional test that compared them. The same is true for 102 and 024 (inscription ##215). (PDF) [file pone.0269544.s004.pdf]

Inscription	Sign Position	Published reading	Reference published reading	Proposed correction (Valério 2013, 2016)	Test Result	P-value
##011. ENKO Abou 010	r00.1	101	Olivier (2007)	102	Negative	1.0
##011. ENKO Abou 010	r00.3	013	Olivier (2007)	008	Positive	2.49×10^{-45}
##014. ENKO Abou 013	r00.1	075	Olivier (2007)	073	Positive	8.19×10^{-152}
##025. ENKO Abou 022	r00.3	008	Olivier (2007)	013	Positive	1.20×10^{-78}
##026. ENKO Abou 023	r00.2	075	Olivier (2007)	073	Positive	1.05×10^{-126}
##026. ENKO Abou 023	r00.5	075	Olivier (2007)	073	Positive	6.32×10^{-147}
##034. ENKO Abou 031	r00.2	095	Olivier (2007)	096	Positive	6.76×10^{-26}
##046. ENKO Abou 043	r00.2	068	Olivier (2007)	097	Negative	1.01
##052. ENKO Abou 049	r00.2	068	Olivier (2007)	097	Negative	1.0
##063. ENKO Abou 060	r00.2	087	Olivier (2007)	088	Positive	5.54×10^{-43}
##095. ENKO Apes 001	r00.7	064	Olivier (2007)	037	Negative	1.0
##098. KALA Arou 001	r15.4	070	Olivier (2007)	087	Positive	4.82×10^{-166}
##108. ENKO Avas 001	r00.2	006	Olivier (2007)	009	Positive	7.96×10^{-39}
##111. ENKO Avas 004	r00.4	008	Olivier (2007)	013	Positive	2.84×10^{-55}
##123. IDAL Avas 001	r00.3	068	Olivier (2007)	097	Negative	1.0
##157. MARO Avas 001	r00.7	068	Olivier (2007)	097	Negative	0.52
##179. CYPR Mvas 002	r00.4	008	Olivier (2007)	013	Positive	1.60×10^{-47}
##194. CYPR? Psce 002	r00.1	068	Olivier (2007)	097	Positive	1.0
##207. ENKO Atab 002.B	r10003d.10	072	Olivier (2007)	070	Positive	0.97
##207. ENKO Atab 002.B	r10004d.10	049	Olivier (2007)	052	Positive	1.40×10^{-5}
##208. ENKO Atab 003.A	r05d.1	005	Olivier (2007)	004	Positive	2.53×10^{-147}
##211. RASH Aéti 002	r00.3	064	Ferrara (2013)	086	Positive	1.90×10^{-9}
##211. RASH Aéti 002	r00.3	064	Ferrara (2013)	112	Positive	3.10×10^{-7}
##211. RASH Aéti 002	r00.3	086	N/A	112	Negative	1.0
##212. RASH Atab 002	r02.3	102	Olivier (2007)	104	Positive	9.01×10^{-61}
##215. RASH Atab 004.A	r08.1	103	Olivier (2007)	102	Positive	1.99×10^{-13}
##215. RASH Atab 004.A	r08.1	103	Olivier (2007)	102	Positive	6.13×10^{-67}
##215. RASH Atab 004.A	r08.1	102	N/A	024	Positive	4.75×10^{-78}
##215. RASH Atab 004.A	r10.2	008	Olivier (2007)	013	Positive	-3.50×10^{-76}
##215. RASH Atab 004.B	r12.3	072	Olivier (2007)	073	Negative	1.0
ADD##229. ENKO Mins 004	r00.2	027	Ferrara (2013)	025	Negative	1.0
ADD##233. IDAL Avas 003	r00.5	025	Ferrara (2013)	027	Negative	1.0
ADD##237. KITI Avas 021	r00.1	006	Ferrara (2013)	102	Negative	1.0
ADD##237. KITI Avas 021	r00.2	023	Ferrara (2013)	061	Positive	1.42×10^{-102}
ADD##242. SANI Avas 001	r00.2	082	Ferrara (2013)	053	Positive	1.70×10^{-51}
