# Supplementary material for: Unsupervised deep learning supports reclassification of Bronze age cypriot writing system
Source: PLoS One. 2022 Jul 14;17(7):e0269544. doi: 10.1371/journal.pone.0269544 (PMC9282481; doi:10.1371/journal.pone.0269544)
Supplement: S5 Table — The correct targets are marked in bold. (PDF) [file pone.0269544.s005.pdf]

Other sign	First 10 *Tablet* signs ranked by distance									
	1	2	3	4	5	6	7	8	9	10
001	**001**	006	005	008	009	040	075	004	076	092
Cosine Distance	**0.07**	0.14	0.39	0.42	0.43	0.53	0.55	0.59	0.61	0.62
004	005	**004**	040	006	001	044	008	029	028	075
Cosine Distance	0.07	**0.13**	0.24	0.34	0.47	0.49	0.59	0.60	0.60	0.61
005	**005**	006	040	004	001	009	044	102	059	107
Cosine Distance	**0.05**	0.24	0.28	0.34	0.41	0.49	0.50	0.54	0.55	0.55
006	**006**	005	001	107	009	008	040	104	102	110
Cosine Distance	**0.14**	0.26	0.33	0.41	0.45	0.48	0.49	0.50	0.58	0.59
008	**008**	001	006	097	096	095	005	029	078	107
Cosine Distance	**0.09**	0.28	0.34	0.40	0.48	0.48	0.50	0.58	0.58	0.58
009	**009**	010	006	027	005	001	059	040	074	060
Cosine Distance	**0.09**	0.23	0.33	0.37	0.38	0.40	0.48	0.51	0.57	0.61
011	**011**	012	005	001	008	040	009	006	028	004
Cosine Distance	**0.33**	0.35	0.42	0.44	0.47	0.48	0.49	0.49	0.50	0.54
012	**012**	028	029	021	033	082	011	008	052	010
Cosine Distance	**0.08**	0.16	0.41	0.42	0.43	0.44	0.51	0.53	0.58	0.59
017	049	080	**017**	052	021	023	029	024	056	051
Cosine Distance	0.23	0.24	**0.26**	0.31	0.37	0.38	0.40	0.42	0.42	0.43
021	**021**	029	028	012	023	024	095	069	072	033
Cosine Distance	**0.15**	0.21	0.32	0.33	0.41	0.42	0.50	0.51	0.54	0.56
023	024	**023**	021	095	072	069	029	033	038	028
Cosine Distance	0.17	**0.24**	0.48	0.50	0.54	0.55	0.57	0.61	0.64	0.65
024	**024**	023	021	033	095	029	028	072	069	012
Cosine Distance	**0.08**	0.24	0.42	0.47	0.49	0.51	0.51	0.56	0.57	0.65
025	**025**	074	110	102	068	072	069	061	064	024
Cosine Distance	**0.09**	0.36	0.39	0.45	0.53	0.63	0.66	0.68	0.72	0.73
027	074	**027**	010	025	072	009	069	089	060	068
Cosine Distance	0.10	**0.22**	0.30	0.43	0.49	0.52	0.54	0.58	0.60	0.61
028	**028**	012	033	010	029	082	021	027	036	008
Cosine Distance	**0.12**	0.15	0.29	0.44	0.51	0.51	0.53	0.54	0.59	0.66
033	024	023	030	**033**	021	029	095	072	069	038
Cosine Distance	0.22	0.29	0.49	**0.50**	0.52	0.56	0.60	0.65	0.65	0.66
036	**036**	035	038	054	037	051	033	052	056	107
Cosine Distance	**0.09**	0.15	0.28	0.32	0.37	0.40	0.49	0.50	0.51	0.63
037	**037**	062	061	059	064	055	044	056	035	060
Cosine Distance	**0.07**	0.12	0.12	0.13	0.15	0.33	0.38	0.45	0.54	0.55
038	**038**	035	036	051	054	056	037	052	024	082
Cosine Distance	**0.19**	0.22	0.31	0.39	0.42	0.44	0.54	0.55	0.60	0.63
044	**044**	062	064	061	059	055	037	060	056	104
Cosine Distance	**0.05**	0.18	0.18	0.26	0.27	0.35	0.40	0.41	0.45	0.47
061	064	062	055	059	**061**	060	090	089	044	087
Cosine Distance	0.17	0.20	0.23	0.27	**0.27**	0.27	0.33	0.35	0.37	0.37

Other sign	First 10 *Tablet* signs ranked by distance									
	1	2	3	4	5	6	7	8	9	10
069	070	071	076	068	073	**069**	075	072	097	095
Cosine Distance	0.25	0.27	0.27	0.29	0.32	**0.36**	0.39	0.40	0.45	0.47
070	073	071	076	**070**	075	092	097	068	100	069
Cosine Distance	0.28	0.35	0.36	**0.40**	0.44	0.48	0.49	0.52	0.53	0.56
075	**075**	087	089	090	070	092	068	076	069	078
Cosine Distance	**0.06**	0.34	0.38	0.39	0.40	0.40	0.41	0.46	0.47	0.48
082	**082**	012	028	052	049	017	051	021	004	029
Cosine Distance	**0.19**	0.22	0.27	0.37	0.43	0.45	0.46	0.46	0.49	0.53
087	**087**	089	090	091	092	060	019	075	027	010
Cosine Distance	**0.16**	0.19	0.23	0.23	0.29	0.30	0.40	0.40	0.50	0.50
096	**096**	095	097	104	068	107	076	082	110	102
Cosine Distance	**0.08**	0.16	0.22	0.41	0.49	0.49	0.52	0.56	0.56	0.56
097	**097**	078	096	079	008	047	095	029	104	004
Cosine Distance	**0.19**	0.37	0.44	0.45	0.48	0.50	0.51	0.57	0.61	0.61
102	104	107	110	**102**	047	079	044	097	078	025
Cosine Distance	0.27	0.39	0.46	**0.57**	0.60	0.60	0.60	0.64	0.67	0.69
104	**104**	052	080	049	004	017	054	044	051	107
Cosine Distance	**0.45**	0.55	0.58	0.59	0.60	0.61	0.61	0.63	0.65	0.66
107	054	104	052	051	**107**	056	049	044	036	080
Cosine Distance	0.30	0.33	0.43	0.45	**0.47**	0.53	0.56	0.58	0.59	0.60
110	104	107	**110**	044	080	054	056	102	079	047
Cosine Distance	0.30	0.45	**0.52**	0.55	0.64	0.64	0.64	0.64	0.66	0.66
