# Supplementary material for: Unsupervised deep learning supports reclassification of Bronze age cypriot writing system
Source: PLoS One. 2022 Jul 14;17(7):e0269544. doi: 10.1371/journal.pone.0269544 (PMC9282481; doi:10.1371/journal.pone.0269544)
Supplement: S6 Table — The correct targets are marked in bold. (PDF) [file pone.0269544.s006.pdf]

| *Other* sign | First 10 *Tablet* signs ranked by distance | | | | | | | | | |
|---|---|---|---|---|---|---|---|---|---|---|
| | 1 | 2 | 3 | 4 | 5 | 6 | 7 | 8 | 9 | 10 |
| 001 | **001** | 009 | 006 | 010 | 027 | 074 | 068 | 092 | 005 | 011 |
| Cosine Distance | **0.09** | 0.11 | 0.28 | 0.36 | 0.38 | 0.38 | 0.46 | 0.48 | 0.52 | 0.55 |
| 004 | 005 | **004** | 040 | 044 | 068 | 006 | 009 | 102 | 059 | 075 |
| Cosine Distance | 0.13 | **0.16** | 0.29 | 0.37 | 0.37 | 0.44 | 0.46 | 0.47 | 0.50 | 0.50 |
| 005 | **005** | 040 | 009 | 006 | 004 | 068 | 001 | 044 | 102 | 110 |
| Cosine Distance | **0.10** | 0.25 | 0.31 | 0.35 | 0.40 | 0.43 | 0.44 | 0.53 | 0.55 | 0.55 |
| 006 | **006** | 009 | 005 | 001 | 068 | 110 | 010 | 025 | 102 | 040 |
| Cosine Distance | **0.20** | 0.30 | 0.34 | 0.42 | 0.42 | 0.47 | 0.48 | 0.49 | 0.50 | 0.50 |
| 008 | **008** | 001 | 009 | 006 | 074 | 069 | 027 | 005 | 072 | 033 |
| Cosine Distance | **0.23** | 0.29 | 0.31 | 0.47 | 0.50 | 0.52 | 0.53 | 0.56 | 0.57 | 0.57 |
| 009 | **009** | 001 | 010 | 027 | 059 | 074 | 005 | 060 | 006 | 012 |
| Cosine Distance | **0.05** | 0.25 | 0.28 | 0.31 | 0.37 | 0.38 | 0.40 | 0.41 | 0.46 | 0.49 |
| 011 | 009 | **011** | 012 | 005 | 040 | 028 | 010 | 001 | 027 | 033 |
| Cosine Distance | 0.27 | **0.29** | 0.36 | 0.36 | 0.44 | 0.44 | 0.46 | 0.50 | 0.54 | 0.55 |
| 012 | **012** | 028 | 029 | 021 | 010 | 027 | 011 | 033 | 009 | 091 |
| Cosine Distance | **0.10** | 0.26 | 0.30 | 0.30 | 0.30 | 0.39 | 0.42 | 0.44 | 0.51 | 0.57 |
| 017 | 080 | 049 | **017** | 052 | 056 | 021 | 051 | 054 | 023 | 047 |
| Cosine Distance | 0.21 | 0.26 | **0.31** | 0.43 | 0.46 | 0.47 | 0.49 | 0.49 | 0.50 | 0.52 |
| 021 | **021** | 029 | 012 | 023 | 075 | 028 | 074 | 024 | 010 | 078 |
| Cosine Distance | **0.11** | 0.13 | 0.35 | 0.38 | 0.40 | 0.47 | 0.50 | 0.52 | 0.57 | 0.58 |
| 023 | 024 | **023** | 021 | 028 | 072 | 095 | 074 | 029 | 033 | 069 |
| Cosine Distance | 0.07 | **0.20** | 0.48 | 0.53 | 0.53 | 0.55 | 0.55 | 0.55 | 0.56 | 0.58 |
| 024 | **024** | 023 | 028 | 033 | 021 | 029 | 095 | 074 | 072 | 035 |
| Cosine Distance | **0.04** | 0.24 | 0.44 | 0.45 | 0.47 | 0.52 | 0.52 | 0.55 | 0.55 | 0.59 |
| 025 | **025** | 074 | 072 | 069 | 076 | 095 | 070 | 027 | 068 | 023 |
| Cosine Distance | **0.22** | 0.29 | 0.44 | 0.46 | 0.49 | 0.51 | 0.52 | 0.54 | 0.58 | 0.60 |
| 027 | **027** | 074 | 010 | 025 | 072 | 019 | 069 | 012 | 009 | 076 |
| Cosine Distance | **0.19** | 0.19 | 0.26 | 0.27 | 0.49 | 0.56 | 0.57 | 0.60 | 0.60 | 0.62 |
| 028 | **028** | 033 | 012 | 024 | 027 | 010 | 036 | 021 | 029 | 082 |
| Cosine Distance | **0.09** | 0.29 | 0.33 | 0.37 | 0.43 | 0.44 | 0.54 | 0.57 | 0.59 | 0.59 |
| 033 | 024 | 023 | **033** | 021 | 029 | 030 | 025 | 074 | 028 | 027 |
| Cosine Distance | 0.19 | 0.21 | **0.49** | 0.50 | 0.54 | 0.54 | 0.55 | 0.56 | 0.65 | 0.66 |
| 036 | **036** | 054 | 035 | 033 | 051 | 049 | 037 | 024 | 028 | 010 |
| Cosine Distance | **0.11** | 0.15 | 0.15 | 0.43 | 0.45 | 0.47 | 0.47 | 0.49 | 0.49 | 0.54 |
| 037 | **037** | 062 | 055 | 061 | 059 | 107 | 019 | 091 | 087 | 010 |
| Cosine Distance | **0.07** | 0.17 | 0.23 | 0.24 | 0.31 | 0.32 | 0.33 | 0.39 | 0.40 | 0.40 |
| 038 | 054 | 035 | 049 | 036 | **038** | 056 | 051 | 052 | 055 | 080 |
| Cosine Distance | 0.26 | 0.30 | 0.31 | 0.34 | **0.38** | 0.43 | 0.45 | 0.48 | 0.48 | 0.50 |
| 044 | **044** | 104 | 062 | 107 | 037 | 059 | 087 | 061 | 110 | 092 |
| Cosine Distance | **0.05** | 0.15 | 0.18 | 0.19 | 0.29 | 0.31 | 0.33 | 0.35 | 0.40 | 0.41 |
| 061 | 062 | 087 | 092 | 044 | **061** | 037 | 055 | 059 | 091 | 090 |
| Cosine Distance | 0.17 | 0.21 | 0.24 | 0.26 | **0.29** | 0.30 | 0.30 | 0.31 | 0.32 | 0.34 |

| *Other* sign | First 10 *Tablet* signs ranked by distance | | | | | | | | | |
|---|---|---|---|---|---|---|---|---|---|---|
| | 1 | 2 | 3 | 4 | 5 | 6 | 7 | 8 | 9 | 10 |
| 069 | 070 | 076 | **069** | 072 | 073 | 075 | 071 | 096 | 097 | 095 |
| Cosine Distance | 0.15 | 0.16 | **0.20** | 0.23 | 0.24 | 0.31 | 0.31 | 0.37 | 0.37 | 0.37 |
| 070 | **070** | 076 | 073 | 069 | 075 | 072 | 071 | 097 | 095 | 060 |
| Cosine Distance | **0.18** | 0.25 | 0.26 | 0.28 | 0.31 | 0.33 | 0.40 | 0.42 | 0.50 | 0.52 |
| 075 | **075** | 076 | 060 | 070 | 069 | 061 | 059 | 089 | 072 | 064 |
| Cosine Distance | **0.04** | 0.22 | 0.25 | 0.28 | 0.29 | 0.35 | 0.35 | 0.36 | 0.37 | 0.42 |
| 082 | **082** | 052 | 049 | 004 | 028 | 087 | 021 | 075 | 029 | 017 |
| Cosine Distance | **0.20** | 0.32 | 0.40 | 0.41 | 0.44 | 0.49 | 0.50 | 0.51 | 0.51 | 0.52 |
| 087 | **087** | 092 | 091 | 090 | 019 | 089 | 059 | 075 | 060 | 021 |
| Cosine Distance | **0.13** | 0.14 | 0.14 | 0.21 | 0.29 | 0.37 | 0.37 | 0.38 | 0.38 | 0.47 |
| 096 | **096** | 095 | 076 | 097 | 104 | 102 | 070 | 051 | 068 | 110 |
| Cosine Distance | **0.10** | 0.25 | 0.43 | 0.45 | 0.48 | 0.49 | 0.53 | 0.54 | 0.55 | 0.55 |
| 097 | **097** | 095 | 096 | 013 | 075 | 004 | 070 | 076 | 069 | 017 |
| Cosine Distance | **0.17** | 0.36 | 0.37 | 0.51 | 0.51 | 0.53 | 0.55 | 0.57 | 0.61 | 0.65 |
| 102 | **102** | 110 | 104 | 044 | 107 | 047 | 068 | 079 | 004 | 038 |
| Cosine Distance | **0.21** | 0.21 | 0.23 | 0.44 | 0.46 | 0.51 | 0.53 | 0.53 | 0.53 | 0.56 |
| 104 | **104** | 044 | 110 | 102 | 004 | 052 | 107 | 056 | 047 | 079 |
| Cosine Distance | **0.20** | 0.36 | 0.36 | 0.36 | 0.47 | 0.47 | 0.49 | 0.51 | 0.55 | 0.57 |
| 107 | 104 | **107** | 044 | 056 | 110 | 052 | 051 | 102 | 038 | 037 |
| Cosine Distance | 0.14 | **0.27** | 0.34 | 0.44 | 0.44 | 0.44 | 0.45 | 0.49 | 0.51 | 0.54 |
| 110 | 104 | 102 | **110** | 044 | 107 | 056 | 047 | 004 | 079 | 038 |
| Cosine Distance | 0.16 | 0.27 | **0.27** | 0.36 | 0.41 | 0.44 | 0.50 | 0.51 | 0.53 | 0.53 |
