# Supplementary material for: Unsupervised deep learning supports reclassification of Bronze age cypriot writing system
Source: PLoS One. 2022 Jul 14;17(7):e0269544. doi: 10.1371/journal.pone.0269544 (PMC9282481; doi:10.1371/journal.pone.0269544)
Supplement: S7 Table — The hypothesized correct targets are marked in bold. Some matches are impossible, because the starting Other sign shape and the target Tablet one are known to coexist in an inscription, where they are contrastive graphemes. Thus, 055 contrasts with both 051 and 095 on clay tablet ##215. 073, 074 and 095 are also contrastive on the same inscription. Impossible matches are discarded and stricken through in the table. (PDF) [file pone.0269544.s007.pdf]

Other sign	First 10 *Tablet* signs ranked by distance									
	1	2	3	4	5	6	7	8	9	10
013	029	074	010	**078**	040	076	095	079	047	080
Cosine Distance	0.32	0.59	0.61	**0.65**	0.81	0.81	0.82	0.89	0.93	1.05
019	010	078	029	**079**	074	090	100	047	062	089
Cosine Distance	0.35	0.52	0.55	**0.59**	0.64	0.65	0.69	0.78	0.79	0.82
034	049	029	054	010	090	051	080	089	040	060
Cosine Distance	0.33	0.39	0.53	0.57	0.61	0.65	0.66	0.66	0.69	0.79
039	**049**	040	080	029	054	090	047	079	078	010
Cosine Distance	**0.41**	0.64	0.65	0.67	0.70	0.72	0.85	0.91	0.92	0.93
041	**010**	074	029	078	079	076	062	090	100	089
Cosine Distance	**0.18**	0.36	0.65	0.84	0.87	0.89	0.93	0.94	0.95	0.96
046	079	**047**	078	080	029	056	040	049	010	062
Cosine Distance	0.35	**0.36**	0.49	0.53	0.57	0.67	0.76	0.88	0.88	0.91
050	080	056	**051**	062	049	090	047	054	079	064
Cosine Distance	0.10	0.13	**0.29**	0.33	0.41	0.45	0.45	0.47	0.48	0.52
053	**054**	051	049	056	080	062	064	090	089	060
Cosine Distance	**0.14**	0.20	0.24	0.26	0.35	0.42	0.52	0.56	0.58	0.68
055	**054**	049	051	056	080	062	090	064	089	010
Cosine Distance	**0.17**	0.37	0.47	0.50	0.58	0.66	0.77	0.78	0.79	0.82
064	**062**	100	090	060	056	064	089	051	054	079
Cosine Distance	**0.27**	0.41	0.47	0.51	0.52	0.53	0.54	0.66	0.74	0.78
073	**076**	074	095	062	060	078	064	079	056	047
Cosine Distance	**0.22**	0.34	0.49	0.55	0.61	0.62	0.64	0.69	0.70	0.71
088	**090**	**089**	060	100	010	064	062	029	076	040
Cosine Distance	**0.20**	**0.33**	0.35	0.54	0.55	0.56	0.57	0.63	0.64	0.65
099	**064**	089	062	060	100	090	076	051	056	054
Cosine Distance	**0.20**	0.23	0.24	0.27	0.31	0.32	0.52	0.58	0.60	0.73
