# Supplementary material for: Unsupervised deep learning supports reclassification of Bronze age cypriot writing system
Source: PLoS One. 2022 Jul 14;17(7):e0269544. doi: 10.1371/journal.pone.0269544 (PMC9282481; doi:10.1371/journal.pone.0269544)
Supplement: S8 Table — The hypothesized correct targets are marked in bold. Impossible matches are discarded and stricken through in the table. (PDF) [file pone.0269544.s008.pdf]

Other sign	First 10 *Tablet* signs ranked by distance									
	1	2	3	4	5	6	7	8	9	10
015	**021**	029	012	~~023~~	028	~~024~~	~~033~~	010	030	~~091~~
Cosine Distance	**0.13**	0.17	0.31	0.39	0.46	0.53	0.56	0.59	0.60	0.64
085	097	095	**096**	~~082~~	075	008	070	076	051	069
Cosine Distance	0.25	0.32	**0.32**	0.46	0.49	0.51	0.53	0.54	0.55	0.57
101	~~104~~	~~044~~	**102**	~~110~~	~~004~~	~~107~~	056	051	068	~~082~~
Cosine Distance	0.15	0.22	**0.35**	0.36	0.41	0.43	0.48	0.50	0.51	0.52
