# Supplementary material for: Unsupervised deep learning supports reclassification of Bronze age cypriot writing system
Source: PLoS One. 2022 Jul 14;17(7):e0269544. doi: 10.1371/journal.pone.0269544 (PMC9282481; doi:10.1371/journal.pone.0269544)
Supplement: Peer review history

# Peer review: Decision Letter 0

**DOI:** 10.1371/journal.pone.0269544.r001

**Authors:** Olivier Morin

1 Nov 2021

PONE-D-21-29213Unsupervised Deep Learning Supports Reclassification of Bronze Age Cypriot Writing SystemPLOS ONE

Dear Silvia Ferrara,

Thank you for submitting your manuscript for consideration at PLOS ONE. I now have in hand reports from two reviewers — one a specialist in computational linguistics and machine learning, the other a specialist of the Cypro-Minoan script with a balanced view of current debates. Both reviewers find merit in this highly original and innovative paper, but recommend that you make extensive changes in order for it to be suitable for publication. I am inviting you to submit a revision which will then be sent back to the same two reviewers. In case the reviews reveal strong disagreements over publication, or new issues, I may contact a third reviewer in addition to those two.

Reviewer 1 provides a great deal of technical advice worth following, especially concerning a relative lack of clarity and exhaustivity in explaining the methods. One concern that I share with them has to do with the validation of your model on cursive Hiragana, which yields lackluster results, calling into question the subsequent applicability of the model to the Cypro-Minoan data. This, in my view, is the number one issue raised by your paper. Please address it in depth, by explaining why the model may yield reliable conclusions in spite of its limited aplicability to a better known and better documented script, and by qualifying your conclusions accordingly.

Like Reviewer 1, I noticed that your paper was not accompanied by open data and code, and that you declared some restrictions would apply to the sharing of the data. Given the highly technical and innovative nature of your study, I do think that giving Reviewer 1 access to your data and code is important to let them appreciate the robustness of the results.

Reviewer 2 confesses serious misgivings about your raw data, but also notes that your conclusion is plausible, and indeed can be defended on other grounds. Please address their thorough and detailed comments; they are mostly dealing with the quality and completeness of the sources. I share their last remark on the fact the three versions of Cypro-Minoan might be one and the same script, without necessarily encoding one and the same language.

Thank you again for allowing us to consider your manuscript.

Olivier Morin

P.S. Please bear in mind this standard caveat if and when you revise the paper: Inviting a revision does not entail that the next version, or any subsequent version, will be accepted for publication. It is my policy to avoid a protracted editorial process that may in any case end in rejection. I am not pre-judging this particular case but this is something I warn all authors of.

Please submit your revised manuscript by Dec 16 2021 11:59PM. If you will need more time than this to complete your revisions, please reply to this message or contact the journal office at plosone@plos.org. When you're ready to submit your revision, log on to https://www.editorialmanager.com/pone/ and select the 'Submissions Needing Revision' folder to locate your manuscript file.

Please include the following items when submitting your revised manuscript:

- A rebuttal letter that responds to each point raised by the academic editor and reviewer(s). You should upload this letter as a separate file labeled 'Response to Reviewers'.
- A marked-up copy of your manuscript that highlights changes made to the original version. You should upload this as a separate file labeled 'Revised Manuscript with Track Changes'.
- An unmarked version of your revised paper without tracked changes. You should upload this as a separate file labeled 'Manuscript'.

If you would like to make changes to your financial disclosure, please include your updated statement in your cover letter. Guidelines for resubmitting your figure files are available below the reviewer comments at the end of this letter.

If applicable, we recommend that you deposit your laboratory protocols in protocols.io to enhance the reproducibility of your results. Protocols.io assigns your protocol its own identifier (DOI) so that it can be cited independently in the future. For instructions see: https://journals.plos.org/plosone/s/submission-guidelines#loc-laboratory-protocols. Additionally, PLOS ONE offers an option for publishing peer-reviewed Lab Protocol articles, which describe protocols hosted on protocols.io. Read more information on sharing protocols at https://plos.org/protocols?utm_medium=editorial-email&utm_source=authorletters&utm_campaign=protocols.

We look forward to receiving your revised manuscript.

[Note: HTML markup is below. Please do not edit.]

Journal Requirements:

When submitting your revision, we need you to address these additional requirements.

1. Please ensure that your manuscript meets PLOS ONE's style requirements, including those for file naming. The PLOS ONE style templates can be found at 

https://journals.plos.org/plosone/s/file?id=wjVg/PLOSOne_formatting_sample_main_body.pdf and 

https://journals.plos.org/plosone/s/file?id=ba62/PLOSOne_formatting_sample_title_authors_affiliations.pdf

2. We note that you have stated that you will provide repository information for your data at acceptance. Should your manuscript be accepted for publication, we will hold it until you provide the relevant accession numbers or DOIs necessary to access your data. If you wish to make changes to your Data Availability statement, please describe these changes in your cover letter and we will update your Data Availability statement to reflect the information you provide

Reviewers' comments:

Reviewer's Responses to Questions

**Comments to the Author**

1. Is the manuscript technically sound, and do the data support the conclusions?

The manuscript must describe a technically sound piece of scientific research with data that supports the conclusions. Experiments must have been conducted rigorously, with appropriate controls, replication, and sample sizes. The conclusions must be drawn appropriately based on the data presented. 

Reviewer #1: Yes

Reviewer #2: No

**********

2. Has the statistical analysis been performed appropriately and rigorously? 

Reviewer #1: Yes

Reviewer #2: I Don't Know

**********

3. Have the authors made all data underlying the findings in their manuscript fully available?

The PLOS Data policy requires authors to make all data underlying the findings described in their manuscript fully available without restriction, with rare exception (please refer to the Data Availability Statement in the manuscript PDF file). The data should be provided as part of the manuscript or its supporting information, or deposited to a public repository. For example, in addition to summary statistics, the data points behind means, medians and variance measures should be available. If there are restrictions on publicly sharing data—e.g. participant privacy or use of data from a third party—those must be specified.

Reviewer #1: No

Reviewer #2: Yes

**********

4. Is the manuscript presented in an intelligible fashion and written in standard English?

PLOS ONE does not copyedit accepted manuscripts, so the language in submitted articles must be clear, correct, and unambiguous. Any typographical or grammatical errors should be corrected at revision, so please note any specific errors here.

Reviewer #1: Yes

Reviewer #2: Yes

**********

5. Review Comments to the Author

Please use the space provided to explain your answers to the questions above. You may also include additional comments for the author, including concerns about dual publication, research ethics, or publication ethics. (Please upload your review as an attachment if it exceeds 20,000 characters)

Reviewer #1: The paper is clearly written and has a straightforward research question, which aims at investigating if three subgroups of the Cypro-Minoan script are the same language or not. The methods used in the paper are relevant for the research question and well described. The appreciation toward the paper is generally positive. However, some revisions could be made to clarify more details, which is why the reviewer suggests: Accept with (major?) revisions.

General comments:

- The title mentions 'deep learning' but within the text, the term changes between 'machine learning' (e.g., also the term ‘machine-based techniques on p2) and 'deep learning'. I suggest to synchronize which term to use when referring to the methods. IMHO, both methods are used in the paper, e.g., k-means is more likely to be affiliated to the machine learning category while neural network is more likely to be affiliated to the deep learning category.

- I understand that the authors have concern to release detailed code and data upon acceptance, but in the current state it is hard to judge how robustly was the analysis conducted. For example, there is not much details about the detailed settings of the parameters and few information from the attached supplementary tables can be used to interpret the robustness of the analysis. The description of the method is well-written though, so the editors may decide if the code is needed for reviewing or not.

- P3: Is there a table or a part of text giving the distribution of the three scripts in the used data? I could not find the information in the text or in the supplementary materials (sorry if I missed it). My follow-up question on the distribution would be: If there is a lack of balance in the data, does this lack of balance between the three scripts have an effect on the output of the experiments?

- P4-P5: “We tested various supervised and unsupervised deep learning models … Our preliminary experiments found ….” If these additional experiments are mentioned, their procedure and output should probably be provided somewhere, either in the text or in the supplementary materials.

- P5: “The model therefore tries to reconstruct the category to which the sign belongs, both from the context (preceding/following sign) and from the sign itself.” This is a cool idea! A quick question though: if the vector model considers the context of each character, isn’t it inherently biased toward a separation of the three scripts? Since only scripts from the same category will occur together?

- P6: for sign2vec, did you consider different window size and type? E.g., three surrounding characters instead of one? Or only considering the words before/after rather than symmetric context? What is the dimension of the output vector? E.g., 50, 100, 500? Sorry if it is already written somewhere and I missed it.

- P6 Table 1: I understand that the authors are considering the Rand index, which is easily affected by the size of different clusters between the predicted and the actual data. Maybe the adjusted Rand index could be considered? Plus, the definition of the metrics listed in Table 1 could be explained. If the journal was a CS or CL journal that might not be necessary, but since PLOS has a larger audience, I suggest to add some brief explanations about those metrics.

- P6 “The scores were not so high because…” If I understand correctly the flow of this section, the authors wanted to validate the model on the Japanese writing system. If the results are not conclusive for Japanese, how do the authors show that the model is reliable?

- P8 “To evaluate our model, we could only use as ground truth a set of 37 signs” I might be a bit confused here. If only those signs were used to evaluate the model, why include the other signs? This is probably already written somewhere in the text but I might have missed it.

- P9 “This demonstrates that, while the vector is not 100% accurate, it is still a reliable method to test the hypothesis that some signs allegedly exclusive to CM1 and CM2 are in reality paleographic variants.” Would it be possible to compare the accuracy obtained here with a random/majority baseline to be able to assess how high or low is the accuracy?

- P11 “These results strengthen the hypothesis that the division of CM in three sub-scripts is invalid, as previously put forward on the basis of paleographic and structural evidence. The implications are of paramount importance for the script,” AFAIU, since the results do not provide a clear-cut (e.g., the accuracy of the models is not very high), I suggest that the authors could be a bit more modest when mentioning the impact of the results. The limitations of the study should also be mentioned somewhere in the conclusion, e.g., the distribution of data? The accuracy of the models and its implication on the interpretability of the results, etc…

Minor comments:

- P1, abstract: “assess if it holds up against a multi-pronged, multi-disciplinary attack”, I suggest to avoid using too strong terms such as 'attack'. However, that might be a personal preference.

- P1: If space allows it, a map showing the location of the sites where the inscriptions have been found could be helpful for readers not familiar with the topic.

- P4: “Almost all neural systems treating images in some ways are based on CNN, thus they seemed most fitting to our ends.” While I agree with the authors, a few references here would be nice to support this statement.

- P5 “we applied some quantitative measures using the MNIST dataset confirming” What are those measures again? I might have missed it.

- P5: I suggest avoiding sentences such as “as mentioned above” in the paper, if you do, please refer to the exact location/section in the text.

- P6: Finally, we combine the DeepCluster-v2 loss, … this is a bit abstract to follow IMHO. Maybe a toy example would help?

- Figure 6 and Figure 7 are hard to interpret visually. Maybe replacing the characters with points and using shapes/colors to distinguish the characters would make it easier to read?

- The format of the refs should be synchronized, for example: [2,15]: The page number seems to be missing, [10,11,30]: The publisher is missing. If the place is required as in [39], it should be added for the other references too.

Reviewer #2: The central question posed in this paper is an old one, and there seems to me some potential to try to address it with new methods of the sort proposed. However, I have serious misgivings about the way in which this research has been conducted. I hope that my specific comments below will demonstrate the grounds for my misgivings, and the reasons why on balance I felt compelled to record that the data (or rather the way in which the data were analysed) do not appear to support the conclusions offered. Unless the authors can address these issues seriously, I fear that the paper comes across as a superficial ‘confirmation’ of pre-existing theories that may otherwise be quite adequately argued via other methods.

P1: The summary of CM inscriptions overlooks at least one further inscription from Tiryns, on the handle of a clay vessel, published by Brent Davis – this work is even on the bibliography (no. 20)! There is also a new potmark from the same site which I believe will be published by the same author.

P3 L64: It seems misrepresentative to say that signs not attested in the tiny repertoire of CM3 were ‘allegedly discarded for linguistic reasons’ (L65). Masson and Olivier both seem to have accepted that there could be signs that simply have yet to be attested in the corpus from Ras Shamra.

It is also worth noting that Olivier was openly sceptical of any linguistic distinction for CM3, making clear in Olivier 2007 that the designation is nothing more than geographical, and often using scare quotes for it (‘CM3’) – even though he maintained Masson’s categorisation.

P4 L106-8: The possibility that some inscriptions at the end of the chronological timespan for Cypro-Minoan might actually be written in the Cypro-Greek syllabary is raised here without any critical commentary on the implications of such an assumption. These documents could be excluded on chronological grounds, but the authors should ideally take some position on their epigraphic status (whether agnostic or not). There have been several recent discussions of the problem, including e.g.:

Duhoux, Y. (2012) ‘The most ancient Cypriot text written in Greek: The Opheltas’ spit’, Kadmos 51, 71-91.

Egetmeyer, M. (2013) ‘From the Cypro-Minoan to the Cypro-Greek syllabaries: linguistic remarks on the script reform’ in Steele, P.M. (ed.) Syllabic Writing on Cyprus and its Context, Cambridge 2012, 107-131.

Egetmeyer, M. (2017) ‘Script and language on Cyprus during the Geometric Period: An overview on the occasion of two new inscriptions’ in Steele, P.M. (ed.) Understanding Relations Between Scripts: The Aegean Writing Systems, Oxford, 108-201.

Steele, P. (2018) Writing and Society in Ancient Cyprus, Cambridge, second chapter.

P4 L111ff: Excluding signs on an essentially linguistic basis is methodologically worrying (the reasoning is repeated on P12). Whether or not there exist arguments in favour of linguistic differentiation, any study of sign shapes / palaeography should be blind to linguistic considerations – which surely is what the authors intend by pursuing the kinds of analysis on offer in this paper.

There might be some sense in excluding all the material from Ras Shamra simply on the grounds that writing practices at that site could be somewhat different from those on Cyprus – though, on the other hand, this might be a good reason for including them. But it must be all or nothing, and the methods employed here cannot seriously investigate the possibility or otherwise that CM3 should be considered as a separate entity from the rest of the CM corpus if tablets #212 and #215 are excluded (and along with them, six sign shapes thus not represented among the data used for this study).

P4 L124ff: Given the aim to achieve more neutral analysis of palaeographic variation in Cypro-Minoan, it is a shame that the authors used published drawings, presumably largely from Olivier where some examples could be criticised as to their representation of features. Those drawings also tend to flatten some kinds of variation owing to palaeographic factors, such as the comparative width of strokes*. Perhaps it is impossible for the present study, but the results of ongoing scanning projects could be particularly beneficial to this kind of analysis because of their more accurate measurement of sign features. There is nevertheless a risk here that the results of the analysis will be affected by pre-existing assumptions and biases on the part of the person who drew the signs, given that any drawing is already in itself interpretive.

*Considerations of this kind indeed seem to have affected the analysis given the divergent clustering of signs on clay documents and signs on other supports, as noted by the authors at P8-9.

P9 L316-8: “This property supports the argument that CM2 is not a script distinct from CM1, but rather a form of the same writing system that differs mainly due to the use of a different writing medium as well as scribal style (smaller and more angular signs).”

This seems to me to be quite a bold claim (not that CM2 is not a separate script in its own right, which is surely at some level true, but that the present investigation can be used as evidence for such a position). I am not convinced that the results can only be read in this way. For one thing, it may be that the quite consistent way in which CM2 signs were drawn (presumably by Olivier?) predisposed them to a differential analysis by the neural network – as I mentioned above, this is a serious risk to the results of the study and needs to be considered carefully.

It would also be helpful to know to what extent differences of scale have been factored in. The signs of the CM2 tablets are far smaller than signs on many other supports, and this makes a difference a) to what it was possible for the author to render, and b) to the accuracy of any modern drawing of the signs. Published editions tend to flatten the degree of difference in size between signs in different inscriptions, but this could indeed be a significant factor in their recognisability (whether to ancient humans or modern computational methods).

P10-11: In the section ‘Application of the Vector’, it is clear that the authors seem to have drawn conclusions that supported pre-held beliefs, but very little information is given as to how the conclusions are supported. Accuracy levels such as 6/10, 7/10, 2/3, 3/3 need to be explained in some detail – what exactly is denoted by these numbers, and what does ‘accuracy’ mean here? Have the results been tested for statistical significance?

P12 L449-451: “If the inscriptions in our dataset (mainly CM1 and CM2) represent the same script, then the likelihood increases that this single script recorded the same language.”

This is an extremely bold and methodologically unsound claim. There are countless examples across the world and across different time periods of different languages being written in a single script / writing system. The language-related considerations offered here do not seem appropriate to the purposes of the paper.

**********

6. PLOS authors have the option to publish the peer review history of their article (what does this mean?). If published, this will include your full peer review and any attached files.

If you choose “no”, your identity will remain anonymous but your review may still be made public.

**Do you want your identity to be public for this peer review?** For information about this choice, including consent withdrawal, please see our Privacy Policy.

Reviewer #1: No

Reviewer #2: **Yes: **Philippa M. Steele

[NOTE: If reviewer comments were submitted as an attachment file, they will be attached to this email and accessible via the submission site. Please log into your account, locate the manuscript record, and check for the action link "View Attachments". If this link does not appear, there are no attachment files.]

While revising your submission, please upload your figure files to the Preflight Analysis and Conversion Engine (PACE) digital diagnostic tool, https://pacev2.apexcovantage.com/. PACE helps ensure that figures meet PLOS requirements. To use PACE, you must first register as a user. Registration is free. Then, login and navigate to the UPLOAD tab, where you will find detailed instructions on how to use the tool. If you encounter any issues or have any questions when using PACE, please email PLOS at figures@plos.org. Please note that Supporting Information files do not need this step.

# Author comment: Author response to Decision Letter 0

**DOI:** 10.1371/journal.pone.0269544.r002

12 May 2022

We thank the reviewers for their invaluable feedback. Thanks to their comments, the revised manuscript has improved both in the experimental settings and the content in numerous ways. Due to the sheer amount of feedback from the editor and reviewers, we provide a detailed response to each question in a separate file included in the revised submission.

Attachment Submitted filename: Response to Reviewers.pdf Click here for additional data file.

# Peer review: Decision Letter 1

**DOI:** 10.1371/journal.pone.0269544.r003

**Authors:** Olivier Morin

24 May 2022

Unsupervised Deep Learning Supports Reclassification of Bronze Age Cypriot Writing System

PONE-D-21-29213R1

Dear Dr. Ferrara,

We’re pleased to inform you that your manuscript has been judged scientifically suitable for publication and will be formally accepted for publication once it meets all outstanding technical requirements.

Within one week, you’ll receive an e-mail detailing the required amendments. When these have been addressed, you’ll receive a formal acceptance letter and your manuscript will be scheduled for publication.

An invoice for payment will follow shortly after the formal acceptance. To ensure an efficient process, please log into Editorial Manager at http://www.editorialmanager.com/pone/, click the 'Update My Information' link at the top of the page, and double check that your user information is up-to-date. If you have any billing related questions, please contact our Author Billing department directly at authorbilling@plos.org.

If your institution or institutions have a press office, please notify them about your upcoming paper to help maximize its impact. If they’ll be preparing press materials, please inform our press team as soon as possible -- no later than 48 hours after receiving the formal acceptance. Your manuscript will remain under strict press embargo until 2 pm Eastern Time on the date of publication. For more information, please contact onepress@plos.org.

Kind regards,

Olivier Morin

Academic Editor

PLOS ONE

Additional Editor Comments (optional):

Dear author,

I am happy to tell you that I am accepting your paper, which both reviewers found to be much improved as a result of this round of revisions. This is an extremely exciting and well designed piece of research which I am certain will move debates forward in several fields.

Thank you for considering PLoS

OM

Reviewers' comments:

Reviewer's Responses to Questions

**Comments to the Author**

1. If the authors have adequately addressed your comments raised in a previous round of review and you feel that this manuscript is now acceptable for publication, you may indicate that here to bypass the “Comments to the Author” section, enter your conflict of interest statement in the “Confidential to Editor” section, and submit your "Accept" recommendation.

Reviewer #1: (No Response)

Reviewer #2: All comments have been addressed

**********

2. Is the manuscript technically sound, and do the data support the conclusions?

The manuscript must describe a technically sound piece of scientific research with data that supports the conclusions. Experiments must have been conducted rigorously, with appropriate controls, replication, and sample sizes. The conclusions must be drawn appropriately based on the data presented. 

Reviewer #1: Yes

Reviewer #2: Yes

**********

3. Has the statistical analysis been performed appropriately and rigorously? 

Reviewer #1: Yes

Reviewer #2: Yes

**********

4. Have the authors made all data underlying the findings in their manuscript fully available?

The PLOS Data policy requires authors to make all data underlying the findings described in their manuscript fully available without restriction, with rare exception (please refer to the Data Availability Statement in the manuscript PDF file). The data should be provided as part of the manuscript or its supporting information, or deposited to a public repository. For example, in addition to summary statistics, the data points behind means, medians and variance measures should be available. If there are restrictions on publicly sharing data—e.g. participant privacy or use of data from a third party—those must be specified.

Reviewer #1: Yes

Reviewer #2: Yes

**********

5. Is the manuscript presented in an intelligible fashion and written in standard English?

PLOS ONE does not copyedit accepted manuscripts, so the language in submitted articles must be clear, correct, and unambiguous. Any typographical or grammatical errors should be corrected at revision, so please note any specific errors here.

Reviewer #1: Yes

Reviewer #2: Yes

**********

6. Review Comments to the Author

Please use the space provided to explain your answers to the questions above. You may also include additional comments for the author, including concerns about dual publication, research ethics, or publication ethics. (Please upload your review as an attachment if it exceeds 20,000 characters)

Reviewer #1: I thank the authors for making the revisions and providing the data and code. I think that the authors did a really good job updating the paper. The provided code also has a clear documentation. I suggest that the paper can be published.

If the editors think that the following minor comments are relevant, they can be transmitted to the authors. If not, the editors may choose to ignore these comments.

Minor comments:

p4 "a recent publication has wanted to see this shape in a new inscription from Erimi-Kafkalla, so we had to count it for our purposes." -> which publication? please add the reference.

Fig 3 -> the circles are bit hard to read and two plots take quite a lot of space by conveying partially overlapping information. Could they be merged? e.g., could we have numbers printed on the circles on the map?

Fig 6 and Fig 7 -> I suggest to put the link to the live 3D scatter plot in the captions, so that is easier for readers to find it.

p7 : "We trained 20 different models initialized with different random parameters. By applying this procedure, the model becomes less susceptible to the the random initialization of its parameters. ": why 20 random parameters? why not e.g., 50 or 100? Please develop a bit how 20 random parameters are excepted to cover the space of randomness for the parameters. I totally understand that there is technical limitations too, as running the code takes time. So, it would not be a problem to mention technical limitations of time, but at least how the number 20 was chosen should be clear.

p7 "The 2560-dimensional representation obtained by applying the proposed neural model, namely Sign2Vecd, and the baseline, DeepClusterv2, was the starting point for any further processing and paleographic consideratio": the use of DeepClusterv2 with the term baseline is not explained before and suddenly shows up here. I suggest to add a sentence or two (here or in the previous text) mentionning why DeepClusterv2 is the baseline. It can be understood from the context, but it always better to make it clear for readers.

Reviewer #2: I would like to thank the authors for their careful consideration of comments from both reviewers. I felt that all my comments had been addressed satisfactorily, and I think that the article now reads very well. I am happy to recommend it for publication without further modifications.

**********

7. PLOS authors have the option to publish the peer review history of their article (what does this mean?). If published, this will include your full peer review and any attached files.

If you choose “no”, your identity will remain anonymous but your review may still be made public.

**Do you want your identity to be public for this peer review?** For information about this choice, including consent withdrawal, please see our Privacy Policy.

Reviewer #1: No

Reviewer #2: **Yes: **Philippa M. Steele

# Editor report: Acceptance letter

**DOI:** 10.1371/journal.pone.0269544.r004

**Authors:** Olivier Morin

22 Jun 2022

PONE-D-21-29213R1 

Unsupervised Deep Learning Supports Reclassification of Bronze Age Cypriot Writing System 

Dear Dr. Ferrara:

I'm pleased to inform you that your manuscript has been deemed suitable for publication in PLOS ONE. Congratulations! Your manuscript is now with our production department. 

If your institution or institutions have a press office, please let them know about your upcoming paper now to help maximize its impact. If they'll be preparing press materials, please inform our press team within the next 48 hours. Your manuscript will remain under strict press embargo until 2 pm Eastern Time on the date of publication. For more information please contact onepress@plos.org.

If we can help with anything else, please email us at plosone@plos.org. 

Thank you for submitting your work to PLOS ONE and supporting open access. 

Kind regards, 

PLOS ONE Editorial Office Staff

on behalf of

Dr. Olivier Morin 

Academic Editor

PLOS ONE
